# Textile Hybrid Electronics for Multifunctional Wearable Integrated Systems

**DOI:** 10.34133/research.0779

**Published:** 2025-07-24

**Authors:** Zimo Cai, Kangjie Ye, Huayu Luo, Jian Tang, Geng Yang, Haibo Xie, Huayong Yang, Kaichen Xu

**Affiliations:** ^1^ State Key Laboratory of Fluid Power & Mechatronic Systems, School of Mechanical Engineering, Zhejiang University, Hangzhou 310058, China.; ^2^ Institute of Advanced Machines, Zhejiang University, Hangzhou 311106, China.

## Abstract

Textiles with intrinsic breathability and mechanical adaptability are revolutionizing wearable electronics by delivering outstanding comfort and integration potentials. However, the expanding functionalities and body coverage of textile electronics necessitate large-scale and distributed driving circuits, which typically compromise the flexibility and permeability of textiles. To overcome the limitations of traditional circuit boards, textile hybrid electronics (THE) have emerged as promising electronic platforms, where both flexible and rigid components are seamlessly integrated into textiles. This route offers a synergy of system-level performance and wearing comfort. The progress report presents a comprehensive overview of the fabrication strategies for fully integrated THE, mainly encompassing the cross-scale production of flexible components and the reliable integration of rigid components. First, typical materials classified in textile electronic components are summarized. Then, various methods for constructing flexible coatings and fibrous structures are elucidated with distinct mechanisms and advantages for textile substrates. Followed by that, the focus shifts to the heterogeneous integration of rigid components, including the layer-by-layer and in-fiber strategies. Both approaches show promise in realizing monolithic and untethered THE systems. Furthermore, representative paradigms of THE are presented for their applications in pervasive health management and human–machine interaction. Finally, future trends of THE toward distributed and intelligent systems are specially emphasized, with key challenges and potential solutions outlined. The combination of various forms of THE is expected to embed advanced electronic functionalities into everyday textiles, bridging the gap between wearable electronics and human life.

## Introduction

Textiles, known for their breathability and exceptional mechanical properties, have become one of the most promising materials for wearable electronics [1,2]. Textile electronics, which refer to textiles with electronic functionalities, were previously a simple combination of off-the-shelf electronic systems and conductive yarns on textile substrates [3]. Such early form of textile electronics was not user-friendly, owing to the rigid and bulky characteristics of traditional electronic systems. Over the past 2 decades, the rapid advancement of flexible electronics has combined diverse fabrication methods with traditional fabric production techniques [4], empowering the current form of textile electronics [5,6]. Today, textile electronic devices not only are composed of various materials but also feature various structures, such as one-dimensional (1D) coaxial structures [7,8], 2D planar structures [9,10], and 3D buckle structures [11,12]. These devices have demonstrated remarkable versatility and impressive performance, achieving breakthroughs in fields such as sensing, energy storages, light emission, and communication [8,13–17]. Moreover, they are being applied to a wide range of body parts, including the head [18], limbs [19], and torso [10].

The evolution of textile electronic devices raises higher demands for their driving circuits, particularly in 2 key aspects. First, larger circuit areas are required to support high-density logic resources for the signal conditioning, computation, and data storage in complex scenarios [20,21]. Second, driving circuits should be distributed across the whole body to shorten analog signal lines, therefore minimizing noise interference [16,22]. Nevertheless, these circuits are typically manufactured on rigid and inextensible printed circuit boards (PCBs). When worn in large-area and distributed configurations, these PCBs restrict body movements and may even cause discomfort or injury. Although circuits on elastic polymer substrates offer an alternative [23,24], they are impermeable, hindering perspiration and potentially causing skin irritation during prolonged uses [25]. As a result, integrating all electronic components into textiles in a ductile and breathable manner has become a key trend in the development of textile electronic systems [26]. Currently, the general electronic components that make up driving circuits still possess rigid mechanical properties. Therefore, the anticipated textile electronic systems will feature the seamless integration of both rigid and flexible electronic components.

**Fig. 1.**
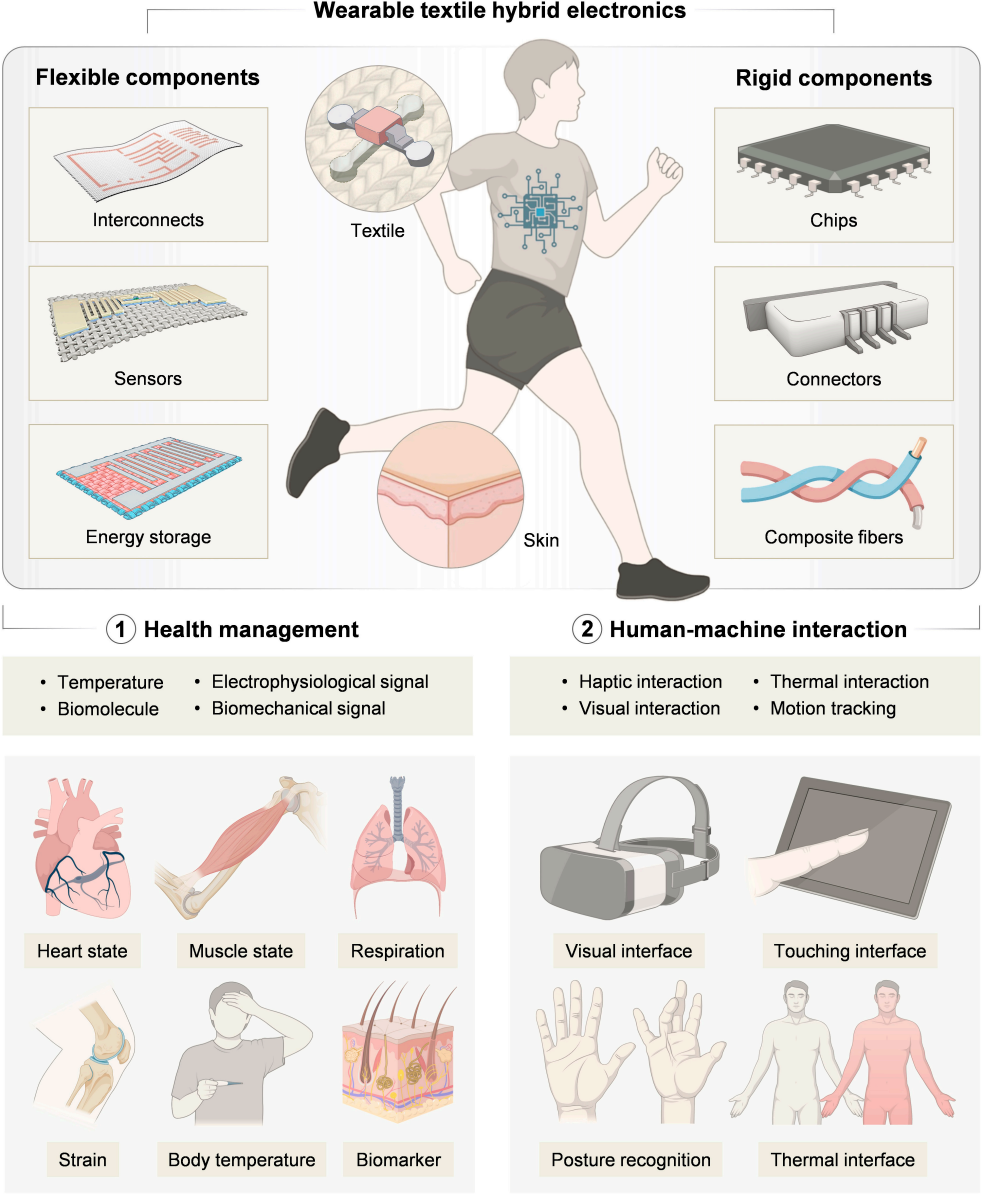
Illustrations of the construction and applications of wearable textile hybrid electronics (THE), which comprise both flexible and rigid electronic components. The flexible components mainly involve interconnects, sensors, and energy storage devices. The rigid components mainly incorporate chips, connectors, and composite fibers. The THE integrated with both flexible and rigid electronic components in a heterogeneous manner is applied in health management and human–machine interaction, functioning as the bridge between wearable electronics and real life. Some figure elements were created using BioRender.com.

Here, we introduce the concept of textile hybrid electronics (THE), as heterogeneously integrated electronic systems on textiles that incorporate both flexible and rigid components (Fig. 1). Flexible electronic components are made from materials with a relatively low modulus, or designed with linear or film-like geometries [27]. These components include conductive interconnects, sensors, energy storage devices, light emitters, and actuators. Rigid electronic components, by contrast, consist of materials with a relatively high modulus and are often characterized by bulky or heterogeneous structures [28]. Common examples include chips, logic units, and connectors.

**Fig. 2.**
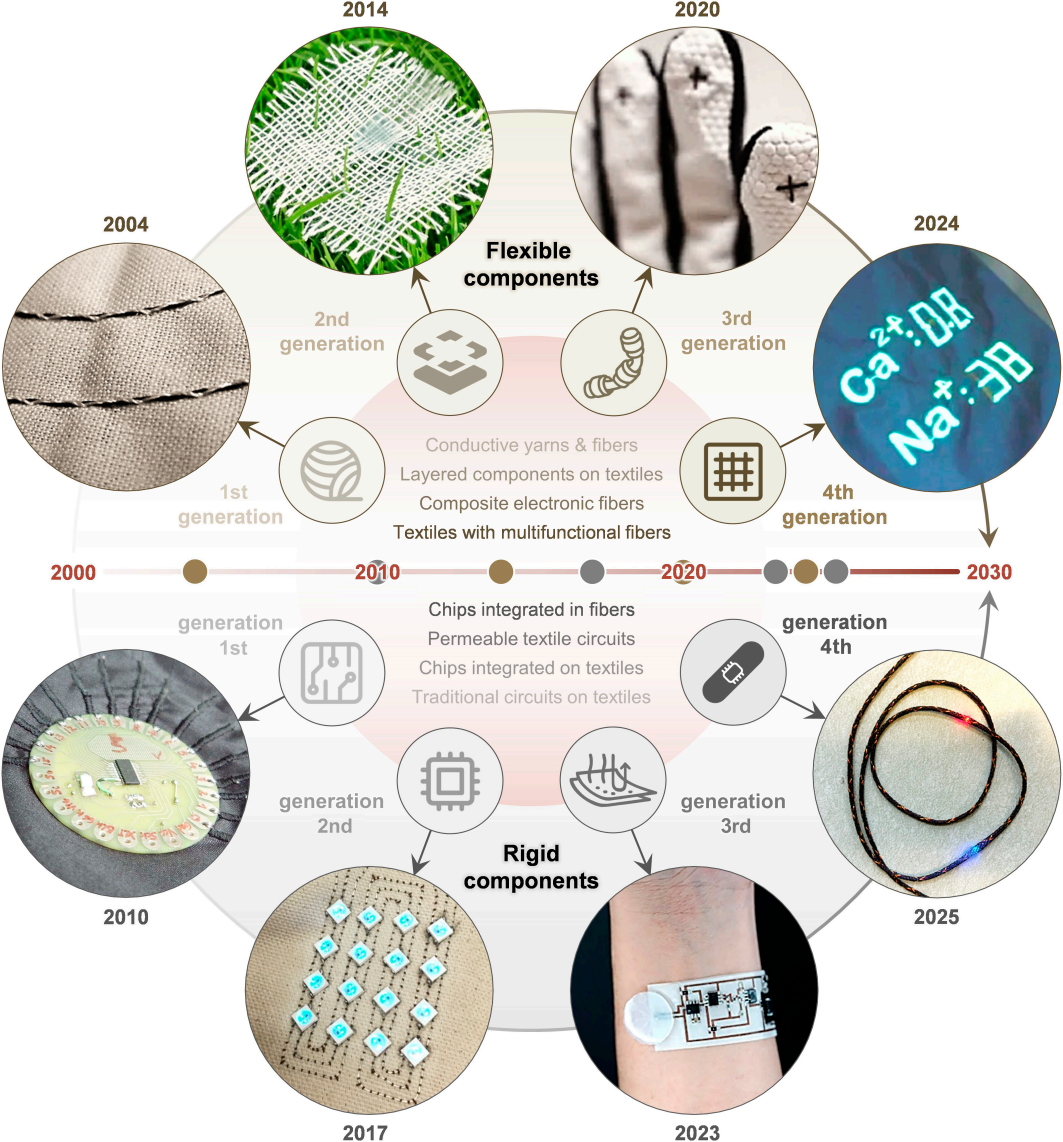
A timeline map of the technological evolution of THE. Flexible textile electronic components are advancing toward multifunctionality, while rigid textile electronic components are evolving to provide a more comfortable and imperceptible wearing experience. An example photo of the first generation of flexible components. Reprinted with permission from [29]. Copyright 2004, Elsevier B.V. An example photo of the second generation of flexible components. Reprinted with permission from [30]. Copyright 2014, Wiley-VCH. An example photo of the third generation of flexible components. Reprinted with permission from [31]. Copyright 2020, Springer Nature. An example photo of the fourth generation of flexible components. Reprinted with permission from [32]. Copyright 2024, Springer Nature. An example photo of the first generation of rigid components. Reprinted with permission from [33]. Copyright 2010, Association for Computing Machinery. An example photo of the second generation of rigid components. Reprinted with permission from [34]. Copyright 2017, Association for Computing Machinery. An example photo of the third generation of rigid components. Reprinted with permission from [19]. Copyright 2023, American Association for the Advancement of Science (AAAS). An example photo of the fourth generation of rigid components. Reprinted with permission from [35]. Copyright 2025, Springer Nature.

The development of both flexible and rigid textile electronic components has substantially promoted the evolution of THE (Fig. 2). Flexible components have advanced in functional complexity. Initially, they were composed of woven or knitted conductive yarns used to form basic devices such as antennas and electrophysiological electrodes [29]. Later, the advancement of printable electronic materials enabled the construction of layered components on fabrics [30]. In the past decade, composite electronic fibers have emerged [31]. These fibers deliver electronic functionality at minimal sizes and enable the fabrication of multifunctional electronic textiles [32]. Meanwhile, rigid components with universal functions have gradually become more flexible and permeable. Conventional PCBs [33] are increasingly replaced by textile substrates [34], with circuitry becoming more complex [19]. In recent studies, chips and wires have been integrated into a single fiber [35]. Hence, these versatile flexible components and imperceptible rigid components together lay the foundations for THE.

Compared with conventional textile electronics, the uniqueness of THE lies in 2 aspects: (a) Heterogeneous components are directly integrated into fibers or textiles, minimizing or even eliminating conventional circuit boards, and (b) all components work together to exchange digital information with other electronic systems or directly interact with humans. Hence, THE represents a category of wearable electronics that combines breathability with system-level functionality. However, formidable challenges remain in the fabrication of THE. First, cross-scale patterning of multiple materials should be achieved on textiles, ranging from tiny footprints of microelectronics to large-area interconnects [36,37]. Second, rigid components should be reliably interfaced with ductile textiles to ensure mechanical and electrical integrity during bending and stretching [38].

**Fig. 3.**
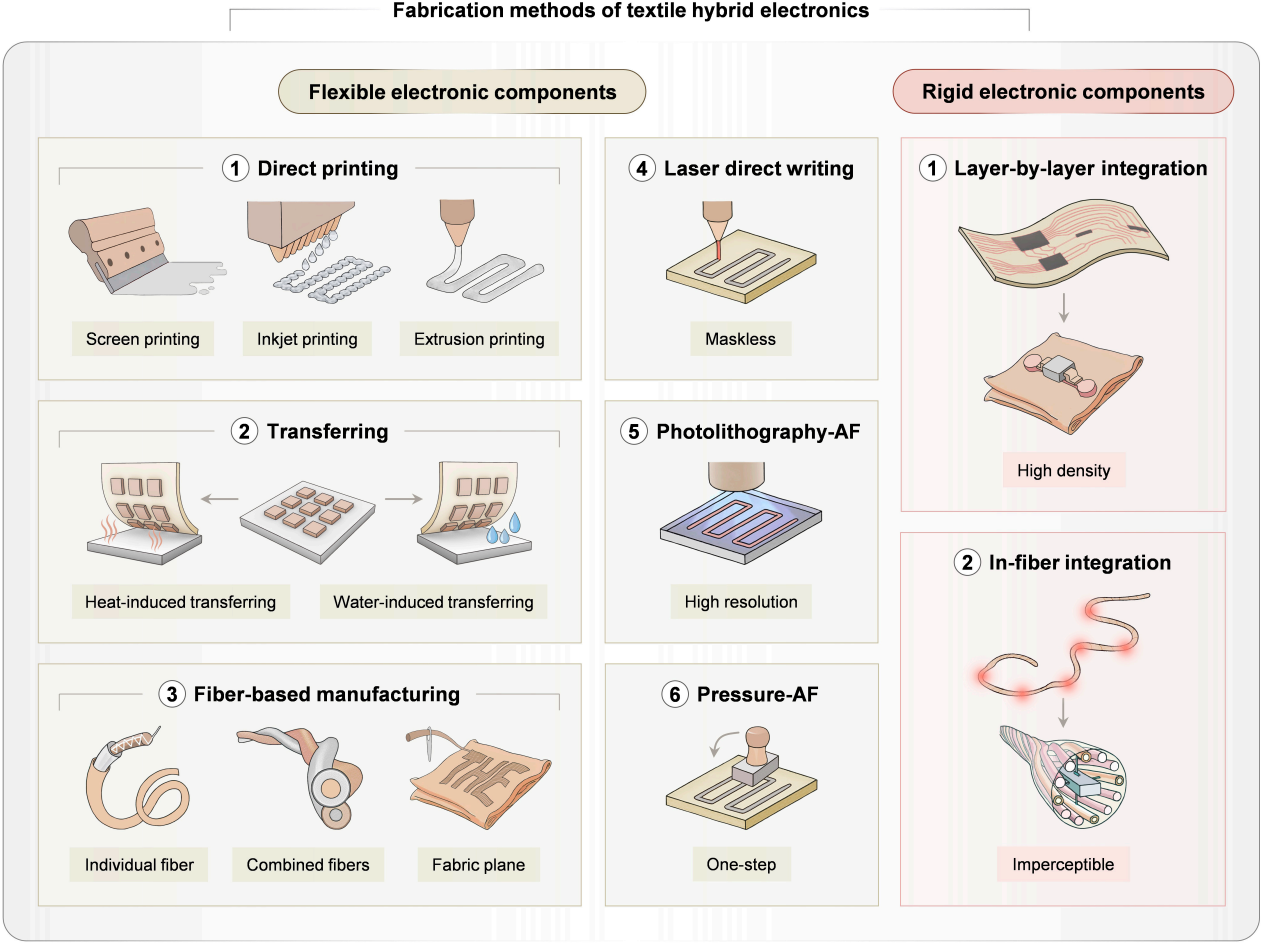
Summaries of representative fabrication methods for flexible and rigid electronic components in THE. The routines for flexible textile electronic components include direct printing, transferring, fiber-based manufacturing, laser direct writing, photolithography-assisted fabrication (Photolithography-AF), as well as pressure-assisted fabrication (Pressure-AF). The routines for rigid electronic components rely on LBL integration and in-fiber integration.

This review focuses on the fabrication of THE, with a particular emphasis on 2 main constituents: flexible and rigid textile electronic components (Fig. 3). First, typical materials for textile substrates and flexible and rigid components are categorized, featured by their distinct properties when functioning in various textile devices (Table). Then, the universal fabrication methods for flexible textile electronic components are discussed, including direct printing, transferring, and fiber-based manufacturing. Direct printing and transferring, originally developed for thin-film electronics, have been continuously refined and are now highly effective for flexible textile components [39]. Fiber-based manufacturing is rooted in traditional textile industry practices. It integrates specialized materials and fiber structures to produce multi-functional textile electronics that offer an imperceptible wearing experience [40]. Furthermore, this review explores other emerging fabrication methods for flexible textile electronic components, including laser direct writing, photolithography-assisted fabrication, and pressure-assisted fabrication. In contrast to flexible components, rigid electronic elements are typically off-the-shelf chips. Fabrication efforts focus on incorporating them into textiles using layer-by-layer (LBL) or in-fiber approaches. We analyze the advantages, limitations, and potential applications of these techniques. Compared with other reviews on textile electronics [41–44], this review starts from the system architectures containing both flexible and rigid electronic components, with a specific focus on the most recent advances in manufacturing and integration processes. We also discuss the latest application paradigms of THE in ubiquitous health management and human–machine interactions, with special emphasis on the edge intelligence embedded in THE for these applications. Finally, we outline the future development trends for THE, summarizing key challenges and potential solutions.

**Table.** Representative materials involved in textile electronic components

Components	Functions	Materials		Features	Effects	References
Textile substrates		Synthetic	Nylon	Ductile, low cost	Daily wearing	[46]
			Kevlar	High strength, stable	Protective clothing	[47,48]
		Natural	Cellulose	Degradable	Eco-friendly fabrics	[51]
Flexible components	Interconnects	Metal	Cu	Low cost	Large-area circuits	[19,53]
			Ag	Stable, antibiotic	Biocompatible devices	[54,55]
		Alloy	LM composite	Fluidic	Stretchable devices	[56,57]
	Sensors	Carbon		Stable, low cost	Strain sensors, pressure sensors	[48,58]
		PEDOT:PSS		Biocompatible	ECG/EMG sensors	[47]
		MXene		Highly sensitive to strain	Pressure sensors	[59,82]
		Ionic TPU		Ionic conductivity	EDL-based sensors	[60]
		FEP		Triboelectric effects	Self-powered tactile sensors	[61]
		Ge		Photoelectric effects	PPG sensors	[18]
	Energy storage	Electrode of batteries	Zn–air	Moderate reactivity	Secure	[62,63]
			Li–O_2_	High energy density	Miniaturized size	[64,65]
			Ca–O_2_	Divalent ion, moderate reactivity	High theoretical capacity, secure	[17]
		Electrode of supercapacitors	Graphene	High surface area, stable	High power density, long lifespan	[67]
			MOF	High surface area, tunability	High power density, programmable chemistry	[69]
	Light emitting	ZnS		Stable, low cost	Large-area	[20]
		ZnS:Cu/BaTiO_3_		Stable, high efficiency	High brightness	[70,71]
Rigid components	Solder	Bi alloy		Solid, low melting point	Strong connection	[72]
		Hybrid LM		Fluidic, adhesive	Stretchable interface	[73–75]
Encapsulations		Polycarbonate		Thermoplastic	Conformal	[76,77]
		Electrospun SBS		Porous	Permeable, waterproof	[73–75]

## Typical Materials for THE

This review first summarizes the representative materials involved in textile electronic components (Table) before elaborating on fabrication techniques. The choice of materials largely determines the applicable fabrication processes and substantially influences the functionalities of individual electronic components as well as the overall system performance.

For textile substrates, their biocompatibility should be first considered to avoid harm or irritation. Generally, hydrophilicity, porosity, and smoothness contribute positively to the biocompatibility of textiles [45]. Moreover, adequate ductility is essential to minimize motion-induced discomfort, while chemical stability is crucial to prevent harmful degradation over prolonged use. Nylon is commonly used as daily fabrics for their low cost and mechanical flexibility, making them well suited for close-to-skin wearable electronics [46]. Kevlar, a representative of protective textiles, offers high mechanical strength, excellent thermal stability, and chemical resistance [47,48]. These properties position it an ideal substrate for electronic systems intended for harsh environments. Recent studies have also proved the biocompatibility of Kevlar [49]. However, both categories of textiles are predominantly synthetic polymers. As such, their textiles are typically nonbiodegradable and may introduce environmental pollution during production. In contrast, cellulose is a natural polymer that generates substantially less environmental impact during processing [50] and is biodegradable in natural settings. This makes it a promising candidate for the development of eco-friendly and biocompatible textile electronics [51]. Moreover, cellulose fibers can be endowed with high ionic conductivity and thermoelectric properties with appropriate material modifications [52]. Such enhanced characteristics further expand their application potentials in next-generation wearable and sustainable electronic systems.

For flexible electronic components, a wide range of functions are involved, each relying on distinct materials. Among them, interconnects serve as fundamental building blocks through the transmission of energy and signals. Metals and alloys exhibit electrical conductivities that are 2 orders of magnitude higher than those of carbons and conductive polymers. Therefore, they are the preferred choice for interconnects in hybrid electronics. Copper (Cu) is the most commonly used metal material in electronic products due to its high conductivity and low cost. It is also adopted in large-area fabric-based electronic devices and systems [19,53]. In comparison, silver (Ag) holds superior chemical stability and exhibits antibacterial properties, making it more advantageous for interconnects in skin-contact wearable textile electronics [54,55]. Meanwhile, liquid metal (LM) and its composites feature both metal-like conductivity and fluidity at room temperature. These natures render them ideal candidates for constructing stretchable textile electronic components [56,57].

In textile electronic systems, sensors play a critical role by converting physical, chemical, and electrophysiological stimuli from the human body or environments into electrical signals. This provides abundant information for health management and human–machine interaction, thus acting as essential components of flexible textile electronics. The core of these sensors lies in stimuli-responsive materials. Carbon and its nanomaterials are often employed as the active layers in resistive sensors, where they respond to pressure or strain accompanied by resistance variations [46,48,58]. Compared with metals, they offer both lower cost and enhanced endurance to oxidation, making them promising candidates for electrophysiological sensors. Besides, its higher electrical impedance helps reduce power consumption in resistive sensors. Conductive polymers, represented by poly(3,4-ethylenedioxythiophene):poly(styrene sulfonate) (PEDOT:PSS), inherently combine stretchability and electrical conductivity. These characteristics make them suitable for electrophysiological sensors that collect electrocardiography (ECG) and electromyography (EMG) signals from the skin surface [47]. MXene is appealing for its excellent processability on textiles, resulting in sensors with both clear patterns and interfacial adhesion [59]. These sensors with microscale layered structures demonstrate ultralow pressure detection limits and high linearity. In addition, ionic polymers [60], triboelectric [61], and photoelectric sensing materials [18] can be integrated with fibers or textile architectures to enable real-time health monitoring and interactions with surrounding environments.

Energy storage serves as the foundational power source for wearable textile platforms. Among various options, fiber-shaped batteries have attracted extensive attention due to their structural adaptability and integration potentials. By engineering electrode and electrolyte materials onto fiber surfaces in a tailored architecture, these batteries are capable of delivering stable voltage outputs. To be specific, fiber batteries can be typically classified based on the materials used for the anode and cathode. Zinc (Zn)–air batteries have gained widespread adoption owing to their favorable safety profile and cost-effective compositions [62,63]. Lithium (Li)–oxygen (O₂) batteries, on the other hand, offer an exceptional advantage in terms of high energy density, making them particularly promising for the miniaturization of textile-based power systems [64,65]. However, the flammability of Li poses safety challenges for practical applications. Calcium (Ca)–O₂ batteries also exhibit high energy density. Benefiting from the intrinsic chemical stability and natural abundance of Ca, Ca–O₂ systems hold merits in both safety and cost, positioning them as a compelling candidate for next-generation fiber battery technologies [17].

Supercapacitors, also known as electrochemical capacitors, hold faster charging and longer cycle life than batteries. Among their components, the electrode material plays a pivotal role in determining the overall performance of supercapacitors [66]. Carbon nanomaterials with subnanometer pores provide considerable surface areas for charge storage while remaining cost-effective [67]. MXene, with tunable surface chemistry and metal-like electrical conductivity, is one of the predominant electrode materials for high-efficient supercapacitors [68]. Metal-organic framework (MOF)-derived electrodes exhibit high surface areas and abundant redox-active sites, thereby enabling excellent charge storage capacity as well as cycling stability [69]. These materials are compatible with fabric fabrication processes, holding promising potentials for integration into flexible and wearable energy storage systems.

Light emitter is considered as the core element of visual interaction; its key functional materials are those capable of converting electrical energy into optical energy. For textile-based light-emitting devices, the electroluminescence effect is widely adopted due to its relatively simple structure and low cost. The electrons in emissive materials are excited by an electric field and emit light as they return to a lower energy state. In this context, zinc sulfide (ZnS) is commonly utilized as the emissive material [20]. As a wide-bandgap semiconductor that emits photons through carrier recombination, ZnS typically produces ultraviolet to blue-wavelength light. By depositing ZnS onto fiber surfaces in specific structural configurations and integrating it with other conductive and dielectric materials within textiles, discrete light-emitting pixels can be fabricated. To enhance the luminescence, additional ion doping within the ZnS matrix, such as Cu^2+^ ions, facilitates the aggregation of charge carriers. In consequence, this increases excitation efficiency and emission brightness. Moreover, incorporating ferroelectric materials with high dielectric constants, such as barium titanate (BaTiO₃), can further improve both brightness and stability [70,71]. Reported studies have demonstrated that these materials can be engineered into composite fibers or applied as multifunctional coatings on textile surfaces, exhibiting excellent applicability for practical scenarios.

In terms of rigid electronic components, they are typically commercial silicon chips. Integrating them into a system requires establishing electrical connections between chip terminals and interconnects, which is realized using soldering materials. In the context of textile electronic systems, bismuth (Bi) alloys represent a suitable choice with a melting point below 150 °C. This helps minimize thermal damage to both interconnects and textile substrates [72]. Meanwhile, Bi alloys remain solid at room temperature and exhibit high modulus and resistance to washing. These properties enable the robust and reliable integration of chips onto textiles. However, the resulting soldered interfaces are intrinsically nonstretchable. To address this limitation, hybrid LM offers an alternative solution [73–75]. It is a biphasic mixture comprising LM and its oxide phases. This composition presents strong adhesion to chip terminals and can be wetted by pristine LM. Consequently, hybrid LM forms a robust conductive bridge between chip terminals and LM interconnects that maintains integrity even under mechanical deformation such as stretching.

In addition to the integration of various flexible and rigid components, textile electronic systems also require effective encapsulation. Encapsulation materials should provide excellent electrical insulation. On the one hand, they prevent undesired electrical contact between electronic components and human skin. On the other hand, they serve as protective barriers against environmental contaminants and mechanical damage. Thermoplastics, such as polycarbonate, enable reliable encapsulation for fiber-based electronic systems [76,77]. During the thermal drawing process, polycarbonate shrinks and wraps tightly around the surfaces of electronic components, resulting in a highly conformal encapsulation layer. This effectively prevents relative displacement among different components. However, such encapsulation materials are unsuitable for large-area textile electronic systems, as thermoplastics tend to clog the pores in fabrics, thereby compromising breathability. To achieve permeable encapsulation, electrospinning techniques can be employed to produce porous nonwoven membranes that adhere to the surfaces of textile platforms [73–75]. The materials applied for electrospinning include poly(styrene-block-butadiene-block-styrene) (SBS), styrene-ethylene-butylene-styrene (SEBS), and thermoplastic polyurethane (TPU). Beyond breathability, these porous layers often exhibit hydrophobic properties, which effectively block external contaminants.

In summary, textile electronic components encompass a wide range of material categories and enable diverse functionalities. This diversity necessitates the development of versatile fabrication techniques capable of processing these materials into specific textile electronic devices and systems.

## Fabrication Methods for Flexible Textile Electronic Components

### Direct printing

The direct printing process deposits liquid or semi-solid materials directly onto textile substrates. Whether followed by postprocessing steps or not, these materials form flexible electronic components [78]. This approach is advantageous due to its short processing time and mild environments. By tuning the physical properties of the ink (e.g., viscosity and surface tension), printing can accommodate a wide range of materials, including conductors, dielectrics, semiconductors, and their fixed forms [79]. However, direct printing on textile substrates is, to some extent, restricted. For low-viscosity inks, the inherent capillary effect of textiles causes the liquid to infiltrate into textiles and spread beyond the printed area, leading to blurred edges and pattern distortion [80]. This issue is particularly pronounced in contact-based printing methods, such as screen printing and stencil printing. The pressure exerted by the printing tools in these methods exacerbates ink infiltration, thereby compromising the fidelity of printed features. High-viscosity pastes or semi-solid inks are more reliant on contact-based printing [56]. For instance, by adjusting the rheological behavior of printed materials, silver traces [81] and MXene electrodes [82,83] can be deposited on fibrous substrates with a resolution as fine as 1 mm, presenting a facile fabrication approach. Despite the fact that their limited fluidity minimizes the influence of capillarity, the rough surfaces of textiles reduce the effective contact area between the ink and substrate. Consequently, greater printing force should be exerted to achieve satisfactory deposition. In such cases, printing fine features, which requires stencils with a high aspect ratio, is challenging. Therefore, direct printing techniques on textiles are better suited for applications where high resolution is not critical and rapid prototyping is desired [84]. The adhesion between the printed coatings and textile surfaces is another focus.

**Fig. 4.**
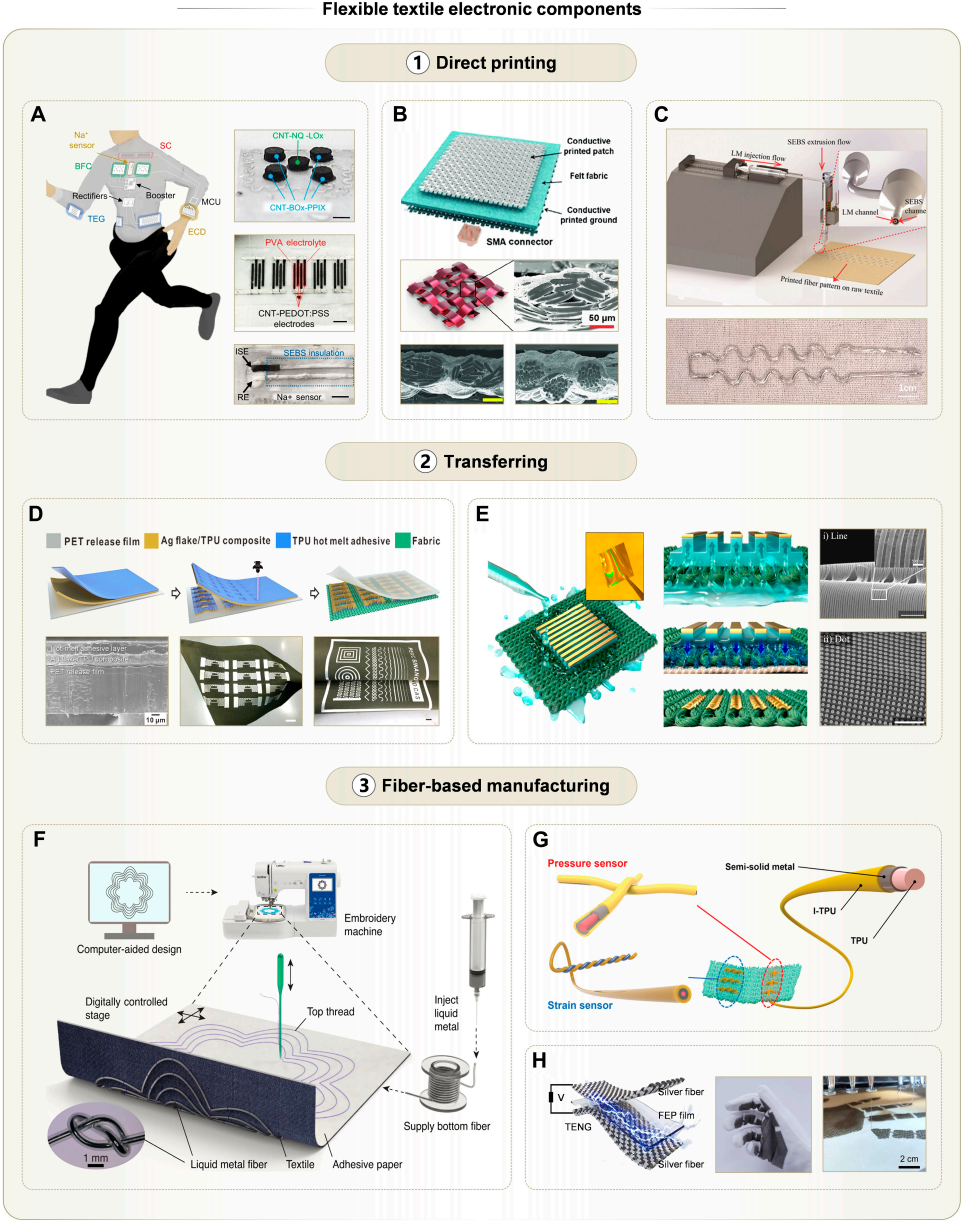
Fabrication methods of flexible textile electronic components and their examples, including direct printing, transferring, and fiber-based manufacturing. Direct printing: (A) Self-powered garment with printed electronic components. Left: Schematic of the garment. Right top: Photo of the biofluid cell. Scale bar, 5 mm. Right middle: Photo of the printed super capacitor. Scale bar, 5 mm. Right bottom: Photo of the all-printed flexible potentiometric sensor. Scale bar, 2 mm. Reprinted with permission from [54]. Copyright 2021, Springer Nature. (B) Printed Ag coatings on woven textiles. Top: Schematic of a patch antenna consisting of Ag coatings printed on textiles with flat yarns. Middle left: Schematic of the woven structure of flat yarn textiles. Middle right: Scanning electron microscopy (SEM) image of the flat yarn textile with printed Ag coatings. Bottom: SEM images of printed Ag coatings on flat (right) and round (left) yarn textiles. Scale bars, 100 μm. Reprinted with permission from [55]. Copyright 2020, Wiley-VCH. (C) Co-printed coaxial fibers on textiles. Top: Schematic of the fabrication process of printed coaxial SEBS/LM fiber. Bottom: Photo of a coaxial SEBS/LM fiber with curved shapes printed directly on textiles. Reprinted with permission from [86]. Copyright 2023, Wiley-VCH. Transferring: (D) Thermal transfer of Ag composites on textiles. Top: Schematic of the fabrication of textile electrodes. Bottom left: Cross-sectional SEM image of the bilayer composite film on the release film. Bottom middle: Photo of the radio frequency identification (RFID) antenna array laminated on cotton textiles. Scale bar, 20 mm. Bottom right: Photo of large-sized textile electrodes with various complex patterns under deformation. Scale bar, 10 mm. Reprinted with permission from [92]. Copyright 2022, American Chemical Society (ACS). (E) Water-induced transfer of nanostructures on textiles. Left and middle: Schematics of water-induced transfer. Right: SEM images of transferred gold (Au) nanopatterns including (i) line arrays and (ii) dot arrays. Scale bars, 5 μm. Reprinted with permission from [96]. Copyright 2020, ACS. Fiber-based manufacturing: (F) LM composite fibers for embroidered electronic components. Reprinted with permission from [57]. Copyright 2022, Springer Nature. (G) Crossed and twisted functional fibers for pressure and strain sensors on textiles. Reprinted with permission from [60]. Copyright 2024, Wiley-VCH. (H) Embroidered fibrous planes for self-powered tactile sensors. Left: Schematic showing the textile-based TENG unit. Middle: Photo of such sensors on a haptic glove. Right: Photo showing the process of embroidery. Reprinted with permission from [61]. Copyright 2025, AAAS.

Representative studies have leveraged the broad material compatibility of direct printing. For example, conductive Ag inks, conductive polymers, dielectrics, and triboelectric materials were sequentially deposited via screen printing onto textile substrates to fabricate a large-area, self-powered biosensing garment [54]. This garment consisted of biofluid cells, super capacitors, and potentiometric sodium ion (Na^+^) sensors (Fig. 4A). Among these, printed SEBS layers served as encapsulations, enhancing the mechanical robustness and wash resistance of the multilayer structure. To address ink infiltration induced by textile capillarity, researchers investigated the influence of yarn structure on the uniformity of printed coatings [55]. They found that flat yarns facilitated coatings with lower roughness and porosity (Fig. 4B). With optimized printing parameters, a conductive Ag layer with a surface roughness of Ra = 4.2 μm and a sheet resistance of 16 ± 3 mΩ/sq was achieved. Compared with unoptimized printing, the resulting Bluetooth antenna demonstrated a 76% increase in communication range.

Interfacial adhesion at textile-coating interfaces can be enhanced via chemical reactions. One study pretreated textiles with ascorbic acid solution and placed them onto Cu foil, followed by inkjet printing of silver nitrate (AgNO_3_) [85]. After that, a localized redox reaction formed Ag conductive traces directly on the textile surface. Due to the enhanced adhesion, the resistance changes of printed Ag during bending were almost negligible. Moreover, this method eliminates the need for high-temperature sintering and thus broadens substrate compatibility. All printed coatings discussed above lack encapsulation. Extrusion printing enables the synchronous fabrication of encapsulation and functional materials [86]. For instance, SEBS shells and LM conductive interconnects were co-printed as coaxial fibers directly onto textiles, offering durability under stretching, bending, and touch (Fig. 4C). Nonetheless, printed coatings can obstruct the pores in textiles, reducing breathability. To overcome this, one study printed LM patterns on elastic nonwoven fabrics and then subjected them to repeated stretching cycles [87]. This process fragmented and regrouped LM along fiber orientations, forming a porous, breathable, and conductive coating.

### Transferring

The transfer technique relocates functional coatings from a donor substrate to a receiver substrate. Typically, these coatings are easier to pattern on the donor surface than on the receiver surface [88]. Due to the capillary effect and rough surface of textile substrates, achieving high-precision coatings directly on fabrics is inherently challenging. Therefore, transferring preformed coatings from smooth, solid donor substrates onto textiles is of critical value. Moreover, the transfer process can protect textiles from damage during fabrication. For instance, posttreatments using corrosive solvents or high curing temperatures may dissolve or degrade textile substrates. By selecting donor substrates that can withstand these harsh conditions, coatings can be fully processed before being transferred onto less chemically tolerant textile receivers. Generally speaking, successful transferring requires 2 fundamental conditions [89]. First, the adhesion between the transferred coating and the receiver must be largely stronger than its adhesion to the donor. This condition should be met either naturally or through some form of activation. Second, the activation method must not irreversibly damage either the transferred coating or the receiver. In practice, thermal or aqueous stimuli are typically used to weaken adhesion to the donor or, in some cases, to decompose the donor entirely.

Heat is the most commonly used activation method. For example, heat transfer paper coated with low-melting materials can serve as a donor substrate [58]. Researchers deposited reduced graphene oxide (rGO) on a polyurethane (PU)-coated heat transfer paper and then thermally laminated the stack onto textiles at 185 °C for 30 s. During lamination, the melted PU detached from the paper and infiltrated the rGO–textile interface, forming strong adhesion. As a result, PU and rGO remained intact and firmly attached to the textile after transfer. Similarly, ethylene-vinyl acetate (EVA)-coated heat transfer paper was used to transfer silver nanowires (Ag NWs) onto fabric at 190 °C for 30 s [90]. Although such thermal transfers achieve high fidelity, the thermally sensitive layer (e.g., EVA or PU) remains above the transferred coating, impeding electrical contact and reducing breathability.

To enable residue-free thermal transfers, a heat-insensitive adhesive can be inserted between the coating and the textile to enhance adhesion [91]. In this way, even if the donor or other unwanted materials adhere to the coating, they can be completely removed without detaching the transferred coating. Further improvements can be realized by employing nonadhesive donors like polyethylene terephthalate (PET) films [70,92]. During hot pressing, PET underwent thermal deformation, reducing its adhesion to the coating (Fig. 4D). Meanwhile, the TPU layer beneath the coating softened and penetrated into the textile, establishing strong mechanical anchoring and adhesion. Upon peeling off the PET donor, Ag-based conductive layers and electroluminescent patterns remain adhered to textiles. LM composites are particularly attractive for wearable electronics due to their intrinsic conductivity and ductility. However, the poor adhesion of LM composites on rough textiles poses a challenge [93]. To address this, researchers preprinted a TPU layer on the textile to enhance wettability and adhesion [94]. To define the pattern of LM composites, another carbon toner layer, with complementary pattern to that of the LM composites, was thermally transferred onto the TPU/textile substrate. Owing to the selective wettability of LM to TPU, rather than to the carbon toner, LM composites could be easily but precisely brushed into specific regions. These regions were the toner-free areas of the TPU/textile substrate, allowing the formation of delicate patterns.

Water can also serve as an activation condition, weakening the adhesion between the donor and the transferred coating. Especially for textiles, the capillary effect facilitates the diffusion of water across the coating, which allows a thorough and fast activation. For metal patterns, water transfer avoids the oxidation that may occur during thermal transfer, thus preserving electrical conductivity [72]. In one case, a substrate coated with water-soluble dextran served as the donor for patterning Ag coatings [95]. After a receiver fiber mat was adhered onto the coating, water was applied, dissolving the dextran within a few minutes. This allowed for considerably lower force to peel off the Ag coatings from the donor, which benefits the accurate transfer of fine patterns. Furthermore, the water-soluble material itself can serve as the donor [96]. Nanoscale patterns of metal and silicon dioxide (SiO_2_) were deposited onto a donor substrate made of water-soluble hyaluronic acid (HA). These delicate coatings were placed face down onto the textile, followed by the dissolution of the HA layer (Fig. 4E). As a result, nanoscale dot, line, and mesh structures automatically adhered to the textile without damage.

### Fiber-based manufacturing

Conventional fiber-based manufacturing can integrate functional fibers into textiles to construct flexible electronic components. These methods involve both the fabrication of fibers and their subsequent incorporation into textiles through weaving, knitting, or embroidery. From a structural perspective, these electronic components can be broadly categorized into 3 types: (a) components formed by individual functional fibers [97,98], (b) components based on the contact between a few functional fibers [99], and (c) components composed of conductive planes formed by embroidering numerous conductive fibers [61]. The primary advantages of fiber-based electronics lie in their preservation of local breathability in textiles, enabling a comfortable user experience. Additionally, fabric-based manufacturing is inherently scalable, making it well suited for large-area production. A major limitation, however, is the appreciable mechanical stress exerted on fibers during textile processing, particularly in knitting and embroidery. Thus, the mechanical stability of fibers is critical to the performance of the resulting electronic components [100].

The most common functional fibers are conductive fibers, whose basic role is current transmission. These can be utilized to construct textile antennas for radio-frequency (RF) communication [101]. By optimizing the arrangement of the conductive fibers, improvements in bandwidth, gain, and radiation efficiency were achieved. To enhance the mechanical robustness of textile antennas in practical scenarios, researchers designed and fabricated coaxial fibers composed of the LM core and the elastomer shell (Fig. 4F) [57]. Owing to the exceptional flexibility of this composite material, the embroidered antenna exhibited negligible changes in resistance even after 10,000 folding cycles. This durability allows the antennas to be applied to highly curved body regions such as the neck, waist, and knees. Conductive fibers also serve as electromagnetic coils and interconnects between distributed modules. For instance, embroidered Cu fibers on a nonwoven glove enabled an array of electromagnetic coils, which drove magnetic actuators to deliver multi-point haptic feedback [102]. On the same glove, tactile sensors were fixed and interconnected by embroidered Ag threads, realizing a haptic glove with both sensing and feedback capability.

The functionality of fiber-based electronics can be extended by incorporating multiple active materials into a single fiber. This approach achieves composite fibers capable of detecting motion and light, as well as storing energy [103]. Recently, scalable manufacturing strategies have been introduced for multiple composite fibers [104]. The proposed woven architecture enabled a one-step integration of fiber-based optoelectronic sensors, supercapacitors, transistors, and light emitters. By optimizing weaving speeds and bonding methods, damage to fibers during processing was effectively minimized.

Devices based on individual fibers have limitations. To overcome this, fiber crossbar (FC) structures, formed at the intersection of 2 orthogonally arranged fibers, have emerged as a promising strategy to realize discrete functional nodes on textiles [99]. FC structures can be designed for multimodal sensing, display, and energy storage. For example, crossing Ag fibers with platinum fibers coated with CuZnS films formed resistive memory elements (i.e., memristors) at each intersection [105]. The woven structure alleviated stress concentrations, endowing the fabric-based memristor arrays with excellent mechanical stability. As a result, they were able to retain their performance even under bending radii as small as 0.1 mm. Apart from crossing, twisting 2 functional fibers offers a larger contact area than the point-wise contact, substantially reducing contact resistance [32]. This characteristic is particularly beneficial for lowering the internal resistance of fiber-based batteries. One recent work combined cross and twist architectures to construct multimodal sensing systems [60]. Specifically, the point contact between crossed fibers formed electric double layers (EDLs) for pressure sensing, while the line contact in twisted fibers formed EDLs for strain sensing (Fig. 4G). Given that cross contacts were strain-insensitive and twisted contacts were pressure-insensitive, this hybrid design effectively mitigated the signal crosstalk under complex mechanical loading conditions [106].

By employing embroidery techniques, a large number of conductive threads can be brought into intimate contact, resulting in an electrically continuous conductive surface. For example, embroidered Ag fibers were used as electrode plates in triboelectric nanogenerators (TENGs) [61]. Integrated into gloves, these electrodes harvested mechanical energy when touching external objects to power other on-glove modules (Fig. 4H). Similar fabrication techniques enabled the integration of capacitors, inductors, and interconnects into a garment, forming a complete inductor–capacitor (LC) resonant circuit for wireless and passive on-body sensing [107]. In this system, capacitors were placed at joints to detect motion-induced strains, while inductors enabled the wireless readout of these motion signals through electromagnetic coupling. Embroidered conductive surfaces can also function as waveguides for transmitting wireless signals [10]. By incorporating these embroidered patterns into safety harness, the near-field interactions between wireless signals and the human body reflected cardiopulmonary information. This provides a battery-free health monitoring route without being in contact with bodies. Beyond planar structures, embroidery facilitates the creation of arched electrodes on textiles that are highly sensitive to normal pressures [108]. These height-adjustable configurations enhanced the performance of TENG-based pressure sensors by optimizing their sensitivities.

### Laser direct writing

Laser direct writing utilizes the highly focused energy of lasers to enable processes such as material ablation [109,110], modification [111,112], or sintering [113]. Among these, laser-induced modification and sintering can selectively and precisely trigger physical or chemical transformations in materials. These techniques have found widespread applications in the fabrication of thin-film flexible electronics [114,115]. In recent years, laser direct writing has also been employed in the development of flexible textile electronic components.

**Fig. 5.**
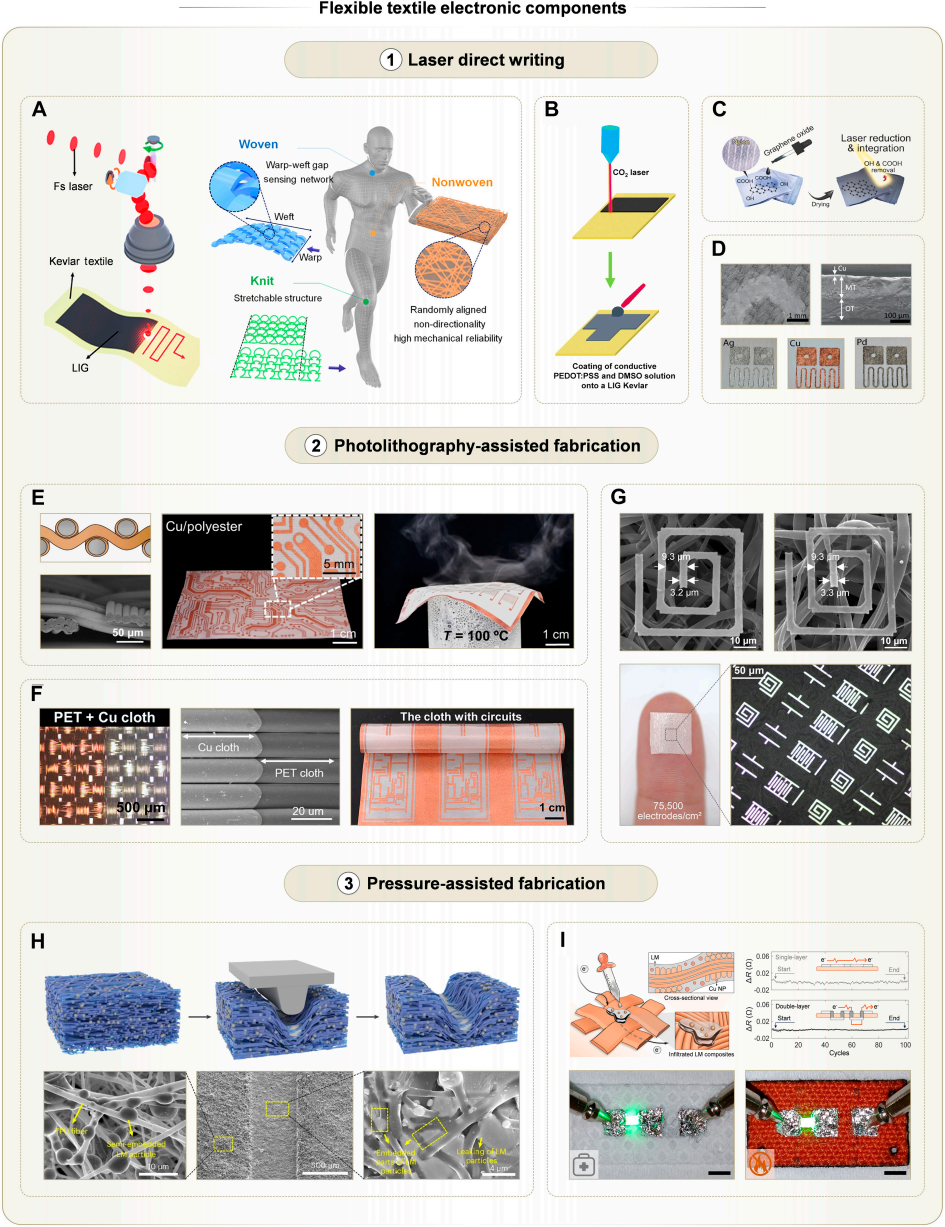
Fabrication methods of flexible textile electronic components and their examples, including laser direct writing, photolithography-assisted fabrication, and pressure-assisted fabrication. Laser direct writing: (A) LIG on various textile structures. Left: Schematic of direct conversion from Kevlar to LIG via one-step maskless femtosecond laser patterning. Right: Schematic of application-specific LIGs and their corresponding textile structures. Reprinted with permission from [48]. Copyright 2023, ACS. (B) Textile-based LIG with polymer coatings as electrophysiological sensors. Top: Laser treatment on Kevlar textile. Bottom: Conductive solution treatment on LIG Kevlar. Reprinted with permission from [47]. Copyright 2023, Royal Society of Chemistry. (C) Schematic showing the fabrication of laser-reduced graphene oxides/textile composite. Reprinted with permission from [46]. Copyright 2023, ACS. (D) Metal films welded on textile surfaces by laser. Top: SEM and cross-section SEM images of metal-textile interfaces on polyethersulfon-cotton fabrics. Bottom: Photos of Ag, Cu, and palladium (Pd) welded on polyamide textiles. Reprinted with permission from [118]. Copyright 2021, Wiley-VCH. Photolithography-assisted fabrication: (E) Left: Cross-sectional schematic and SEM image of Cu patterns fabricated by in-textile photolithography method. Middle: Photo of the Cu PCB circuit patterned in polyester textile, with an inset of the high-resolution image showing the Cu tracks in the textile. Right: Photo showing the water vapor permeability and softness of the Cu-patterned textile. Reprinted with permission from [53]. Copyright 2024, Springer Nature. (F) Left: Photo of the pattern edge of Cu interconnects, showing the sharp edges of patterned metallic textiles enabled by double-sided photolithography. Middle: Cross-sectional SEM image of etched Cu cloth. Right: Photo of large-size PET cloth with complicated Cu-patterned circuits. Reprinted with permission from [19]. Copyright 2023, AAAS. (G) LM micropatterns on nonwoven textiles. Top left: Ag micropatterns on fibrous SBS textiles made by photolithography and water-induced transfer. Top right: LM micropatterns alloyed with Ag. Bottom: Photos of diverse LM micropatterns with high density. Reprinted with permission from [95]. Copyright 2023, AAAS. Pressure-assisted fabrication: (H) Pressure-sintered LM traces on nonwoven textiles. Top: Schematics of the preparation of pressure-stamped conductive path in LM-containing nanofiber membrane (LMNM). Bottom (from left to right): SEM images of the unstamped area, the pressure-stamped conductive path, and the stamped area of LMNM. The semi-embedded LM particles guarantee that the LM is always in a stable combination with the fiber before and after stamping. Reprinted with permission from [121]. Copyright 2024, Springer Nature. (I) Pressure-induced vertical electrical conduction on textiles. Top left: Schematic showing the mechanism of vertical conductive pathways on textiles. Top right: Resistance variations of a single-layer conductive textile wire and a double-layer conductive textile wire under repeated bending. The arrows indicate the start and end points of bending processes. Bottom: Photos of vertical conductive pathways in series with bright light-emitting diodes. Scale bars, 2 mm. Reprinted with permission from [72]. Copyright 2025, IOP publishing.

One representative approach is laser-induced graphene (LIG), typically achieved by irradiating amide-rich precursors with a laser [116]. The interfacial photothermal effect triggers localized carbonization, producing conductive and porous carbon patterns well suited for carbon-based electronics [117]. To be specific, the textile substrate generates accumulated heat through thermal energy conversion from photon energy under laser irradiation. Following this, the lattice vibrations in the carbon-enrich textile are intensified to cause the breakdown of chemical bonds (e.g., C–O, C=O, and C–N) and the recombination of residual carbon. This leads to recrystallization, carbonization, and the ultimate graphitization of textile precursors, along with the emission of gas products. By applying this method to amide-rich textiles, 3D porous carbon patterns can be directly formed on textile substrates. For instance, researchers employed a pulsed laser with a wavelength of 1,035.67 nm and a pulse width of 255 fs to carbonize Kevlar fabric, generating LIG patterns [48]. They further explored how textile structures affect the performance of LIG (Fig. 5A). The nonwoven structure offered the highest mechanical stability, making LIG on such substrates ideal for strain-insensitive applications such as temperature sensors and supercapacitors. The knitted structure exhibited excellent elasticity. Therefore, LIG on prestretched knitted textiles served as strain sensors. The woven structure, due to its distinct sensitivity to mild pressure, was suitable for highly sensitive pressure sensors, such as those for detecting vocal vibrations.

Beyond the bare LIG, LIG can also be functionalized with other materials to develop electronic components. For example, LIG formed on Kevlar fabric was drop-coated with a PEDOT:PSS/dimethyl sulfoxide (DMSO) solution for 2 cycles. The inclusion of PEDOT:PSS imparted LIG with resistance of a few ohms. This LIG/PEDOT:PSS electrode demonstrated its potential for long-term electrophysiological monitoring (Fig. 5B) [47]. To expand the applicability of LIG to a wider range of textile substrates, researchers utilized laser-induced reduction to convert graphene oxide (GO) coating on textiles into LIG [46]. In this case, the GO water dispersion was drop-coated onto textiles and dried, forming a GO layer with a density of 6 μg/mm^2^. The laser energy removed oxygen-containing groups in the GO layer, thereby restoring conjugated graphene structures. This method enabled the formation of robust LIG patterns on amide-free nylon textiles. Such patterns also showed strong resistance to sonication, indicating high reliability (Fig. 5C). Moreover, these LIG patterns modified with laser-reduced silver nanoparticles (Ag NPs) functioned as surface-enhanced Raman spectroscopy (SERS) sensors on textiles, which were capable of detecting glucose in sweat.

Apart from LIG, the laser with photothermal effects also melts metal films on textiles and facilitates strong metal-textile adhesion for robust interconnects and sensors. In one study, researchers employed a 940-nm near-field laser to irradiate metal films coated with a light-absorbing layer [118]. At the focal point, the metal melted, and the spherical focal lens simultaneously applied ~1 bar of pressure (Fig. 5D). The combined heat and pressure melted and embedded a wide range of metals into textile substrates, including Ag, Cu, and Pd. More importantly, the resulting metal coatings with a thickness of less than 1 μm exhibited strong adhesion to textiles. They also withstood up to 10,000 cycles of abrasion under the Martindale test, underscoring their durability for practical applications.

In addition to fabricating flexible components, lasers can also be utilized to modulate the strain distribution around flexible components [119]. Through photothermal effects, the laser controlled the polymerization process and consequently tuned the modulus of the polydimethylsiloxane (PDMS) substrate. When stretching the substrate, its soft regions released strain while flexible sensors located on the stiff areas remained unaffected. Although this strategy has not yet been extended to textiles, it is promising for developing textile electronic systems that are immune to strain interference.

### Photolithography-assisted fabrication

Depositing high-resolution coatings on textile substrates remains a challenge due to their inherent roughness and porosity. As one of the most precise patterning methods, photolithography presents a promising approach for fabricating textile electronic components with high-resolution features.

In a pioneering study, researchers demonstrated the use of photolithography to achieve conductive interconnects with a linewidth of 400 μm on woven textiles [19,53]. The process involved polymer-assisted metal deposition (PAMD), which developed a uniform metal layer over the entire textile through electroless plating. Since the metal finely conformed to the woven structures, the plated patterns preserved the textile’s intrinsic porosity, allowing remarkable permeability. Next, double-sided photolithography was applied to selectively remove metal from designated regions, thereby forming patterned conductive traces (Fig. 5E). Notably, the produced metal traces exhibited clear and smooth boundaries, implying outstanding resolution (Fig. 5F). These permeable and elaborate metal traces could serve as the building blocks of wearable biochemical sensors. However, due to the discontinuous woven structure of textiles, reducing the linewidth below 400 μm severely increased the resistance.

To overcome the limitations of fabric structures on linewidth, metal films with continuous conductive paths are preferred. The same research group employed a photolithographic process to first fabricate ultrafine Ag patterns on a donor substrate coated with water-soluble adhesive [95]. These continuous metal features were then successfully transferred to a nonwoven fabric, enabling Ag patterns with a minimum linewidth of 2 μm (Fig. 5G). To further enhance their electrical performance, LM was selectively applied onto the fine Ag wires, leveraging the strong wettability of LM with Ag. The introduction of LM not only compensated for structural defects in Ag patterns formed during transferring but also preserved conductivity under mechanical deformation such as stretching. Owing to the porous nature of the nonwoven structure, water could easily penetrate through the textile. This process dissolved the adhesive layer on the donor substrate, allowing damage-free and clean transfer of fine patterns. At the same time, the porosity ensured permeability, showcasing promising potential for applications in health monitoring and bioelectronics.

### Pressure-assisted fabrication

Pressure applied to textile substrates can induce structural changes in both textiles and the coatings above. These pressure-induced effects offer opportunities for the fabrication of textile-based electronic components.

One representative study utilized pressure-constrained sonication, which modified universal printed coatings on textile surfaces [120]. Prior to treatment, particles within the coatings were typically encapsulated by polymeric or oxidized layers and exhibited relatively large interparticle spacings. Such coatings lack both mechanical stability and electrical performance particularly on textiles. By exposing these coatings to the sonotrode, acoustic vibration and its thermal effects softened particles. This also disrupted their encapsulating layers, leading to compatible plastic deformation and interparticle bonding. Meanwhile, pressure reassembled these softened particles into a dense accumulation. As a result, these coatings achieved markedly higher conductivity and uniformity, and their adhesion to textile substrates was substantially improved. Using this approach, researchers demonstrated conductive interconnects with a minimum linewidth of 200 μm on textiles. These interconnects were further developed into charging antennas, electrophysiological sensors, electroluminescent devices, and heaters.

Pressure also enabled mechanical sintering of LM particles, forming conductive pathways within the pressed regions. While this principle is well established in thin-film electronics, it has recently been adapted for textile electronics. In one study, researchers formulated a dispersion of LM particles in a TPU matrix and fabricated nonwoven textiles via electrospinning, embedding LM particles within the fibers [121]. By applying localized pressure using a stamp, vertical compression of the fibrous network brought numerous LM particles into contact (Fig. 5H). At this step, LM particles merged and formed in-plane conductive pathways exactly at pressed regions. Moreover, the low wettability of LM to the fibrous substrate prevented it from spreading, contributing to stable LM traces without flowing elsewhere. This method is notable for its simplicity, achieving a minimum linewidth of 50 μm. It also maintains reliable performance under up to 300% strain, making it highly suitable for elastic, breathable electronic circuits.

Pressure can also facilitate the formation of vertical conductive pathways, which are crucial for 3D interconnects and the integration of complex electronic systems on textiles. In one study, pneumatic pressure of 200 kPa was applied to press LM composites on textile surfaces [72]. The surge of pressure ruptured the native oxide layer of LM, instantaneously increasing its fluidity. The LM was then infiltrated into the pores of textiles, forming vertical interconnect accesses (VIAs) (Fig. 5I). These VIAs were tolerant to strain, and thus, double-layer circuits with VIAs exhibited neglectable resistance variations during bending. In addition, this method was compatible with a variety of textile structures, including woven, knitted, and nonwoven. Compared to conventional methods such as perforation or drilling, this approach preserved the structural integrity of textiles, thereby maintaining their mechanical strength.

Overall, each of the aforementioned fabrication techniques holds its own advantages. Methods such as direct printing and transferring are relatively simple, enabling multilayer components on both sides of textile substrates. However, these approaches locally compromise the breathability. In contrast, the components produced via fiber-based manufacturing exhibit discrete structures, thereby preserving the inherent permeability of textiles. Moreover, these electronic components demonstrate stronger anchoring with textile substrates and generally resist machine washing. Therefore, for large-area textile electronic components, fiber-based manufacturing should be prioritized whenever possible.

## Integration of Rigid Electronic Components into Textiles

With flexible electronic components already formed on textiles, rigid components are then integrated together to form a monolithic THE. The integration methods of rigid components fundamentally impact the geometrical and mechanical properties of hybrid systems.

### LBL integration

Rigid electronic components, primarily integrated circuit (IC) chips, are essential to most electronic systems. The concept of LBL integration in THE draws inspiration from traditional PCB assembly [122,123]. In this scheme, rigid components are arranged on one horizontal plane, while the textile substrate and its conductive interconnects lie on another. The interconnects contain the footprints of all rigid components. Electrical connections between each rigid component and its footprint are established using conductive materials.

**Fig. 6.**
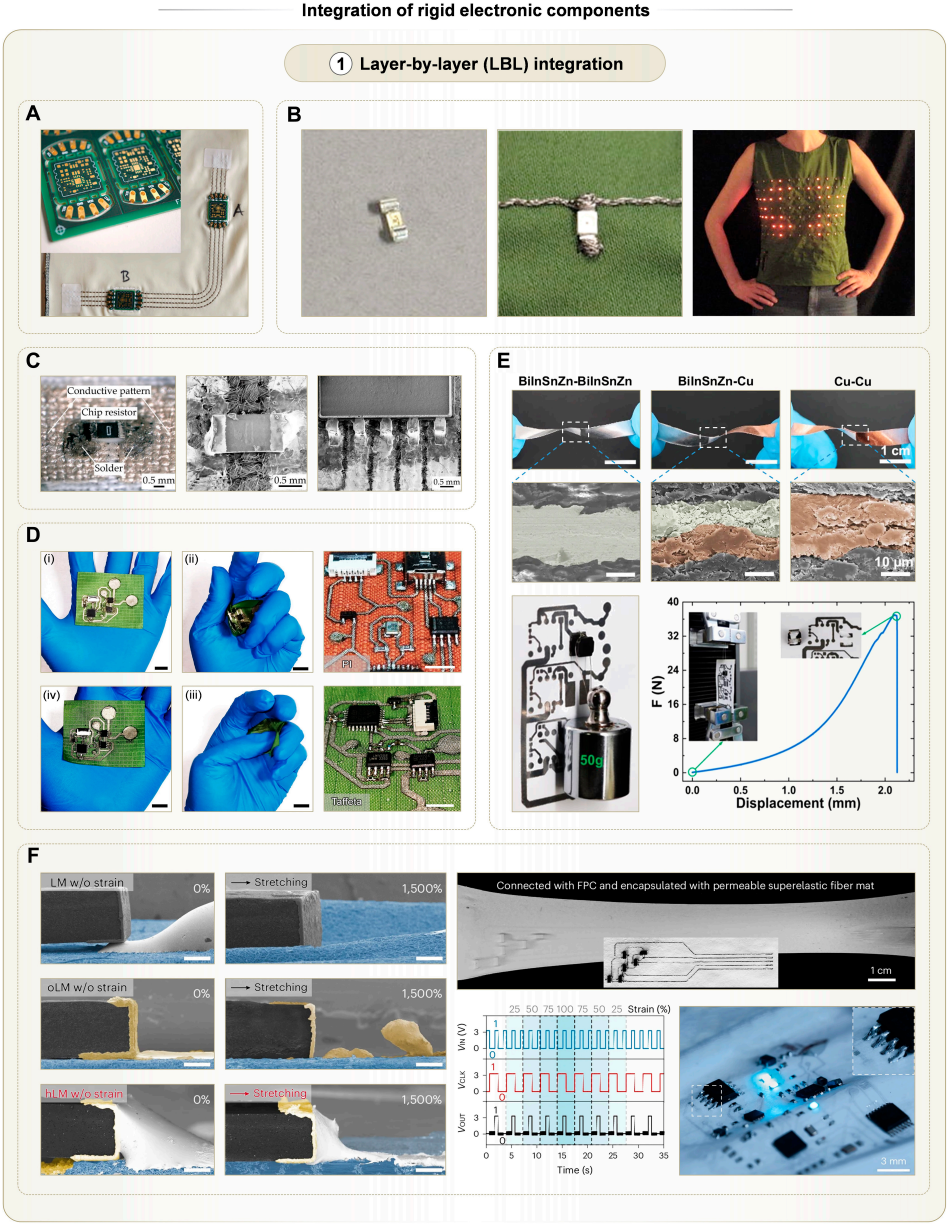
LBL integration strategy of rigid electronic components into textiles, along with some examples. (A) Photo of an interposer sewn on textiles by conductive threads. Reprinted with permission from [127]. Copyright 2025, Wiley-VCH. (B) Chips with crimped terminals that can be soldered on conductive threads. Left: Photo of this type of LED chip. Middle: Photo of the LED soldered on conductive threads. Right: Photo of a wearable LED display mounted in a shirt. Reprinted with permission from [128]. Copyright 2007, Springer Nature. (C) Standard chips directly soldered to printed interconnects on textiles. Left: Photo of a resistor soldered to printed conductive interconnects. Middle and right: SEM images showing solder joints of various chips. Reprinted with permission from [38]. Copyright 2020, MDPI AG. (D) Monolithic ECG sensing system on textiles consisting of soldered chips and transferred interconnects. Left (i to iv): Photos showing the mechanical endurance of the THE sensor subjected to repeated crumpling. Scale bars, 1 cm. Right: Photos showing surface-mount devices (SMDs) soldered on conductive textile wires on 2 textiles. Scale bars, 5 mm. Reprinted with permission from [72]. Copyright 2025, IOP Publishing. (E) Pressure-constrained sonication that bonds chip terminals with interconnects on textiles. Top: Photos of 2 stacked homogeneous (or heterogeneous) printed metal electrodes bonded by the pressure-constrained sonication activation (PCSA) method, including BiInSnZn–BiInSnZn, BiInSnZn–Cu, and Cu–Cu join. Middle: Cross-sectional SEM images of the bonding interfaces. Bottom left: Photo showing that the electronic device strongly bonds with the printed BiInSnZn electrode activated by PCSA. Bottom right: Adhesion force testing of bonding interfaces. Reprinted with permission from [120]. Copyright 2024, Springer Nature. (F) LM hybrid solder that bonds chip terminals with LM interconnects on stretchable substrates. Left: SEM images showing the electrical interfaces of rigid components using pristine LM, oxidized LM (oLM), and hybrid LM (hLM) solder, which exhibits the superiority of hLM solder to LM and its oxides. Scale bars, 200 μm. Top right: Photo showing the stable electrical performance of the stretched textile circuit. Bottom middle: Logic outputs of the circuit during stretching. Bottom right: Photo showing high-density chips integrated by this method. Reprinted with permission from [73]. Copyright 2024, Springer Nature.

In some studies, all rigid components are preassembled onto thin-film substrates, which are then attached to textiles via either adhesive bonding or stitching [124–126]. Although these approaches are straightforward, the resulting circuits severely hinder air permeability of underlying textiles, which deteriorates wearing comfort. As an alternative, interposer technology was introduced. The interposer is a small-area thin-film translation circuit interfacing both rigid components and textiles [127]. The central region of interposers was connected to rigid components, while the surrounding area featured perforated conductive pads (Fig. 6A). Conductive threads embroidered through these holes established connections between textile interconnects and interposers. By decomposing a high-density circuit into several modules on interposers, the breathability and mechanical compliance of the system were improved. However, this approach increased the total area occupied by the circuits. Hence, an ideal LBL strategy should directly connect chip terminals to the textile interconnects, avoiding any intermediate substrates.

LBL integration of rigid components depends on the surface morphology of textile interconnects. For interconnects formed by fiber-based manufacturing, their surfaces are inherently textured due to the fiber structure. As a result, these surfaces have difficulty forming tight contact with planar chip terminals, which may lead to brittle failures at soldered interfaces [34]. To address this issue, researchers developed chips with crimped terminals. These terminals were curved to conform to conductive threads, leading to increased contact areas [128]. This technique rendered sewable chips, and their terminals were wrapped with conductive threads during the integration on fabrics, enabling an interactive light-emitting diode (LED) garment (Fig. 6B). However, chips with such customized terminals are rare, limiting the scalability of this method.

By contrast, textile interconnects fabricated using direct printing or transferring methods tend to be relatively smoother, facilitating direct bonding with standard chip terminals (Fig. 6C) [38]. For instance, an alloy solder with a melting point of 138 °C was used to bond chip terminals to transferred interconnects on textiles [72]. The mechanical robustness of soldered joints enabled a monolithic ECG sensing system that remained functional after repeated rubbing (Fig. 6D). Chips can also be bond onto interconnects using pressure-constrained sonication [120]. This method leveraged the heat induced by acoustic vibration, softening common alloy solders (e.g., BiInSnZn) and Cu at their contact interfaces. Thus, separate metal strips and alloy strips could be tightly bonded (Fig. 6E). Similarly, the interconnects beneath chip terminals were softened, forming strong electrical and mechanical connections with chip terminals after solidifying. These connections at bonding interfaces were strong enough that a 2-terminal chip was capable of withstanding a pulling force up to 36 N. The main advantage of this technique lies in its material simplicity and compatibility with various substrates. Additionally, this method eliminates the need for conductive adhesives or solders at the contact interfaces between chip terminals and interconnects.

All the abovementioned strategies result in rigid electrical connections, which limit the stretchability of THE. To address this, ductile LM has been explored for this purpose. However, the poor wettability of LM with chip terminals often leads to delamination under strain. As a solution, researchers proposed a hybrid LM solder composed of LM and its oxides [73]. The solder material combined the ductility of LM with the adhesiveness of LM oxides, enabling a highly stretchable and adhesive connection between chip terminals and LM interconnects (Fig. 6F). Using this approach, the hybrid circuits on elastic nonwoven textiles achieved electrical integrity even under 1,500% strain, demonstrating prospects for high-density and stretchable THE. However, this solder was not mechanically tough and chips were prone to shift when lateral force was applied. Therefore, additional glues should be applied between chip corners and substrates.

### In-fiber integration

**Fig. 7.**
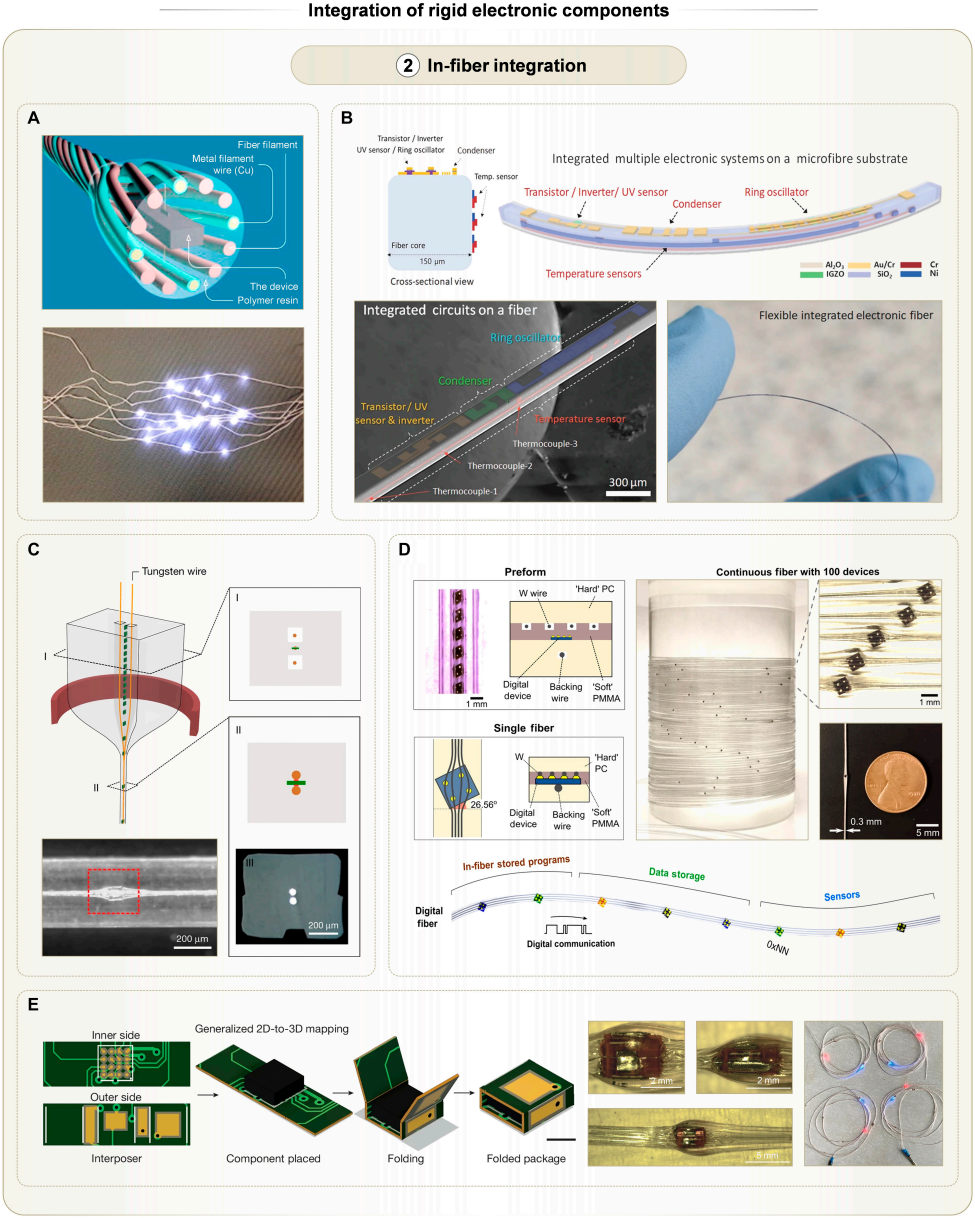
In-fiber integration strategy of rigid electronic components into textiles, along with some examples. (A) Chips embedded in hybrid yarns consisting of conductive fibers and ordinary fibers. Top: Schematic of structural details. Bottom: Photo of LED yarn. Reprinted with permission from [129]. Copyright 2015, Woodhead Publishing. (B) Microfiber with multiple electronic systems integrated using microfabrication. Top: Cross-sectional and 3D schematics of the devices fabricated on the microfiber substrate. Bottom left: SEM image showing structural details of the microfiber. Bottom right: Photo showing the flexibility of the microfiber. Reprinted with permission from [130]. Copyright 2022, Springer Nature. (C) Two-terminal chips embedded in thermally drawn polymer fibers. Top: Schematics showing thermal drawing processes. Bottom left: Photo showing details of an LED chip inside the drawn fiber. Reprinted with permission from [76]. Copyright 2018, Springer Nature. (D) Four-terminal chips embedded in thermally drawn polymer fibers. Top left: Schematics showing cross sections of the preform before drawing and the drawn fiber. Top right: Photos of a continuous drawn fiber with 100 integrated devices. Bottom: Schematic showing various kinds of chips connected in one drawn fiber, performing machine-learning inference. Reprinted with permission from [77]. Copyright 2021, Springer Nature. (E) Multi-terminal chips with 3D interposers embedded in thermally drawn polymer fibers. Left: Schematics showing the capability of 3D interposers, which are folded from planar interposers. Middle: Photos showing details of chips and interposers inside drawn fibers. Right: Photo showing multiple chips embedded in one fiber. Reprinted with permission from [35]. Copyright 2025, Springer Nature.

While LBL integration enables high-density THE, it often introduces localized rigidity and a loss of breathability. This results in a surface profile that is considerably higher than that of pristine textiles, thereby compromising the fabric’s tactile comfort. Embedding rigid electronic components, such as chips, directly into fibers or yarns offers a promising solution for maximizing breathability, ductility, and tactile comfort of THE. In early efforts, researchers embedded LED chips into yarns, which were twisted by ordinary fibers and metal fibers together [129]. Currents flowed through metal fibers into chip terminals, illuminating the LEDs inside yarns (Fig. 7A). However, this integration method was limited to chips with specific packages. To enable the integration of more complex components, microfabrication technologies were employed to construct a variety of semiconductor devices on a 150-μm-wide rectangular fiber [130]. These devices included inverters, ring oscillators, photodetectors, and temperature sensors, achieving system-level functionality within a single fiber (Fig. 7B). Despite the functional diversity, the process complexity hindered large-scale adoption.

To simplify integration, researchers developed a thermal drawing technique, wherein prealigned chips, parallel conductive wires, and thermoplastic polymers were assembled into a fiber precursor, known as a preform. The preform was heated and drawn into elongated fibers [76]. During the drawing process, the preforms shrunk uniformly and the wires were unspooled into hollow channels flanking chips. The lateral separation of wires was gradually reduced in the neck-down region until the electrical contact was established with chips (Fig. 7C). Using this method, double-terminal chips, such as LEDs and photodetectors, were seamlessly integrated into polymer fibers in a single step. This integration enabled functionalities such as optical communication and photoplethysmography (PPG)-based pulse measurement. To extend the thermal drawing technique to chips with more terminals, researchers elaborated the tilt angle of chips as well as a hybrid preform architecture [77]. These allowed the integration of 4-terminal chips and 4 parallel conductive wires in one drawn fiber (Fig. 7D). At this stage, the categories of chips were extended to digital temperature sensors and memory modules. However, the process still exhibited limitations, with a failure rate of chip-wire connection at ~10%, which increased with terminal numbers.

To further increase the quality rate as well as compatibility of thermal drawing, 3D interposers were introduced to bridge a variety of surface mounted chips with parallel conductive wires [35]. In this approach, customized 2D interposers were first soldered with chips and then folded into 3D geometries (Fig. 7E). The geometries of the 3D interposers were designed to align their perforated pads with the layout of parallel conductive wires. With such interposers, chips with arbitrary terminal numbers and arrangements could be converted into a 4-terminal configuration and seamlessly embedded into a single fiber. Using this technique, a fiber computer was developed that integrated microcontrollers, Bluetooth low energy (BLE) chips, and PPG sensors, showcasing immense potentials for wearable health monitoring and human–machine interaction. Due to the limitation in routing noncoplanar pads of 3D interposers with parallel wires, the number of wires in drawn fibers was restricted. Therefore, these fibers are more suited for small-scale and distributed electronic modules. Given the transformative potential of the thermal drawing technique, there is increasing attention on the mechanical stress imposed during the process, which can damage brittle semiconductor materials. To address this, researchers proposed a 2-step thermal drawing strategy informed by mechanical modeling, effectively reducing structural defects in the resulting semiconductor-integrated fibers [18]. This strategy ultimately enabled the fabrication of continuously drawn fibers up to hundreds of meters in length, highlighting the commercial scalability of in-fiber integrated electronics.

Comparing the LBL integration with the in-fiber integration, in-fiber THE offers a balance between wearing comfort and basic computational capabilities, but it faces limitations in circuit layouts and chip sizes. In contrast, LBL THE, despite having greater local rigidity, enables more complex and versatile functionalities. For large-scale textile electronic systems, the combination of both strategies may offer a more advantageous solution.

## Applications of Wearable THE

By integrating both flexible and rigid electronic components into textiles, THE achieves a balance between wearing comfort and system-level functionality. To date, THE has demonstrated application paradigms across various fields, where its core functionalities largely remain within the domains of health management and human–machine interaction. The compact and monolithic structure, combined with artificial intelligence (AI), enables THE to display superior application performance compared to conventional textile electronic systems.

### Health management

#### Epidermal health management

**Fig. 8.**
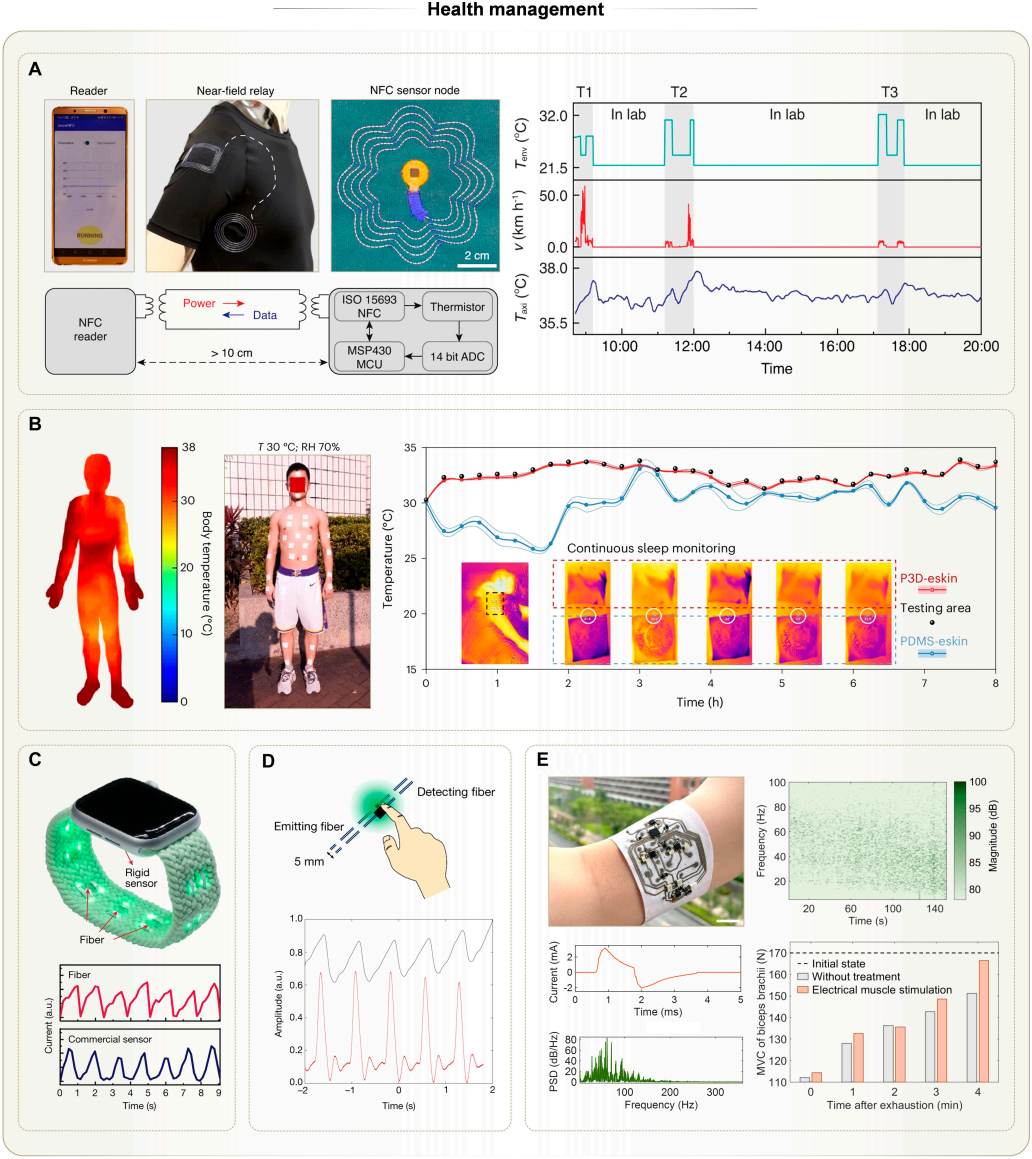
Applications of THE in health management (part 1). (A) Battery-free temperature sensing system by embroidery and the interposer technology. Left top: Photos of the sensing system showing a cellphone reader, an embroidered relay, and a temperature sensing node. Left bottom: Schematic showing the working mechanism of the sensing system. Right: Measured axillary temperatures by the system during a 12-h period, along with environmental temperatures and velocities of the volunteer. Reprinted with permission from [57]. Copyright 2022, Springer Nature. (B) Battery-free and fully integrated temperature sensing node on the stretchable nonwoven textile. Left: Thermal imagery of an adult body wearing 40 such sensing nodes, depicting body temperature in warm and damp environments. Right: Continuous temperature monitoring of an adult body during sleeping using this sensing node, compared with Au standard and another sensing node on the elastomer film. Reprinted with permission from [73]. Copyright 2024, Springer Nature. (C) Watchband with embedded germanium fiber and LED chips for measuring heart pulses via PPG. Top: Photo showing the flexible structure of the watchband compared with a rigid commercial sensor. Bottom: Comparison of measured pulses between the watchband and the commercial sensor. Reprinted with permission from [18]. Copyright 2024, Springer Nature. (D) In-fiber electronics embedded with a photodetector chip and an LED chip for measuring heart pulses via PPG. Top: Schematic showing the measurement setup by placing a finger above both chips. Bottom: Comparison of measured pulses between the in-fiber electronics and a commercial sensor. Reprinted with permission from [76]. Copyright 2018, Springer Nature. (E) Fully integrated textile band with combined monitoring and therapy. Left top: Photo of this textile band worn on the forearm of a volunteer. Scale bar, 2 cm. Left bottom: EMS current delivered by the band and EMG signals measured by the band from biceps. Right top: Frequency shifts of EMG signals in the biceps detected by the band during fatigue. Right bottom: Comparison of the recovery of muscle forces between the control group and the experimental group treated with EMS by the band. Reprinted with permission from [72]. Copyright 2025, IOP Publishing.

As textiles are in prolonged contact with human epidermis in daily life, THE provides a natural platform for long-term epidermal health management, such as monitoring body temperature, hydration, or strain. Among those, body temperature serves as an important indicator of fatigue, exercise intensity, and environmental adaptability [131,132]. For example, researchers developed a wireless and battery-free axillary temperature sensor on textiles using embroidered antennas and interposer technology [57]. To avoid placing a bulky reader under the arm, they designed a near-field relay using embroidered conductive fibers. This relay electromagnetically coupled the underarm antenna to a secondary antenna on the upper arm (Fig. 8A). With this approach, placing the cellphone reader on the upper arm allowed real-time temperature acquisition from the armpit. The system achieved a continuous 12-h monitoring, with reliable performance during movement and perspiration.

To eliminate the use of nonbreathable and nonstretchable interposers, temperature sensors along with related battery-free signal conditioning circuits were fabricated on breathable, stretchable nonwoven fabrics using hybrid LM solders [73]. This system, named permeable 3D (P3D)-eskin, noticeably improved wearing comfort, allowing up to 40 sensors to be simultaneously attached to human skin for multi-point temperature mapping (Fig. 8B). Compared with interposers, the stretchable fabric substrate was more conformable to the skin, improving heat transfer to the skin. Moreover, the permeability of the P3D-eskin kept normal convective heat transfer between air and the covered skin. In this way, the measured temperature was more faithful and reflective of actual skin temperature, showing superiority to its counterpart based on nonbreathable films.

Pressure sensors capable of capturing human vital signals also serve as effective tools for health management. For example, monitoring plantar pressure assists in evaluating training postures during rehabilitation [133]. Using structure-gradient and fibrous triboelectric pressure sensors, dynamic plantar pressure within a wide range (200 to 600 kPa) was successfully detected. An insole embedded with 9 such sensors was developed to map plantar pressure and assess postural accuracy during Bulgarian squats. With the aid of a machine-learning algorithm, the system provided objective and actionable guidance to the user. In the future, hybrid circuits for signal acquisition can be integrated into these sensor arrays, enabling portable and continuous health monitoring.

#### Transdermal health management

THE is also well suited for detecting transdermal physiological signals, which reflect the health status of cardiovascular or muscular tissues. PPG has been successfully implemented in THE for cardiovascular monitoring. One example integrated an LED array and a germanium fiber into a wristband [18]. The 532-nm LED illuminated the wrist, and the reflected light was detected by the germanium fiber, which generated a photocurrent representing the pulse waveform (Fig. 8C). A similar concept was implemented in finger-based sensing [76], using 2 parallel fibers woven into a textile. One fiber was embedded with a 532-nm LED chip, and the other was embedded with a gallium arsenide (GaAs) photodetector chip (Fig. 8D). When a fingertip simultaneously touched both fibers, the photodetector produced an electrical signal synchronized with the heartbeat.

**Fig. 9.**
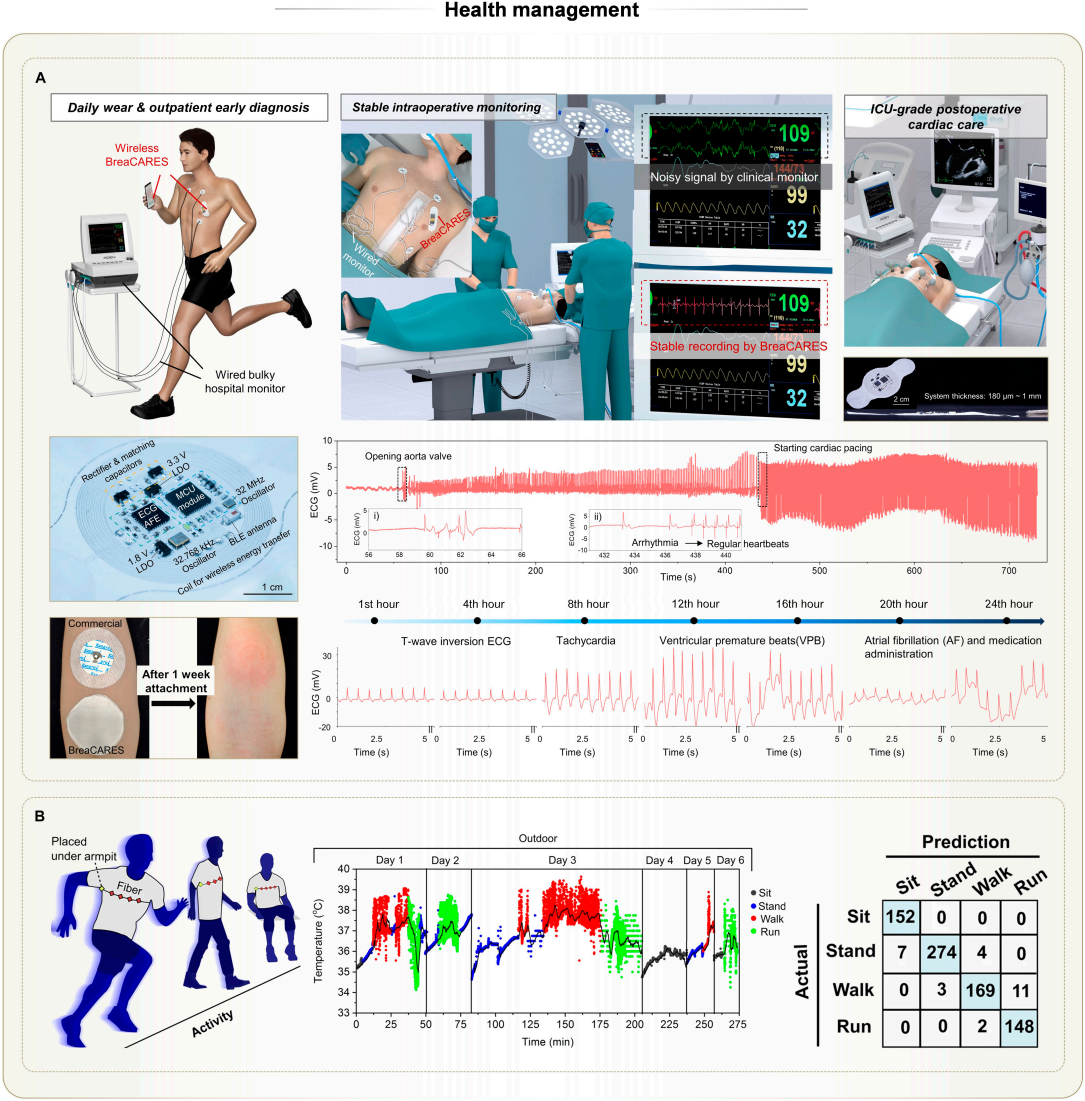
Applications of THE in health management (part 2). (A) Monolithically integrated ECG sensing system with conformal and comfortable biointerface. Top left: Schematic showing the system for wireless and real-time cardiac care in daily scenarios and early diagnosis. Top middle: Schematic showing the system in intraoperative cardiac monitoring of surgical patients. Top right: Schematic showing the system in ICU-grade postoperative cardiac care. Middle left: Photo showing detailed structures of assembled chips on the system. Middle right: Continuous and stable intraoperative ECG signals measured by the system during surgery. Bottom left: Photo showing the inflammation of skin after covering the commercial electrode and the abovementioned sensing system for 1 week. Bottom right: Continuous and real-time postoperative inpatient monitoring by the system in cardiovascular ICU for 24 h. Reprinted with permission from [75]. Copyright 2025, AAAS. (B) In-fiber temperature sensing system performing machine-learning inference. Left: Schematic showing that the system infers human activities based on body temperatures. Middle: Time plots of the in-fabric stored body temperatures across 4.5 h and across different activities. Right: Prediction table of human activities recognized by the in-fabric algorithm, showing an average accuracy of 96.4%. Reprinted with permission from [77]. Copyright 2021, Springer Nature.

Owing to the high-density integration of LBL THE, researchers developed a fully integrated textile platform with combined physiological monitoring and therapy [72]. An EMG sensor, an electrical muscle stimulator (EMS), and their driving circuits were integrated on polyester textiles (Fig. 8E). The circuits were composed of transferred interconnects, press-induced VIAs, and soldered chips. Such a system detected EMG frequency shifts in the biceps during fatigue. In this case, the integrated EMS alleviated muscular fatigue by enhancing blood circulation. This therapy achieved a superior recovery of muscular contraction strength compared with that in a natural rest. Benefiting from the wearing comfort, THE is applicable not only in daily life but also in more demanding clinical settings. Researchers fabricated a battery-free single-lead ECG sensor system on a nonwoven fabric, named BreCARES [75]. The sensor employed LM–hydrogel composite electrodes to acquire ECG signals from human skin. Circuits for power management, signal amplification, and wireless transmission were integrated using the hybrid LM solder (Fig. 9A). Compared with commercial equipment, this monolithic and breathable sensor system offered superior comfort. It also maintained clinical-grade accuracy, even during continuous intraoperative and postoperative monitoring in the intensive care unit (ICU). ECG signals indicating various cardiac arrhythmias and regular pacing were clearly detected.

#### Edge intelligence for health inference

Since THE allows the integration of chips and computation, algorithms can be deployed locally, enabling edge intelligence that infers human behavior. In one study, a digital temperature sensor and several memory chips were serially embedded in one drawn fiber, with a memory density of 760 kbit/m [77]. The system not only recorded continuous temperature data but also stored pretrained neural networks (Fig. 9B). Using the axillary temperature as an input, the local algorithm achieved a 96% accuracy in predicting human activities such as sitting, standing, walking, and running. These edge-deployed algorithms are critical for large-scale, distributed wearable sensing systems, as they help reduce the computational load on central processors and alleviate bandwidth requirements for data transmission between nodes.

### Human–machine interaction

#### Visual interaction

**Fig. 10.**
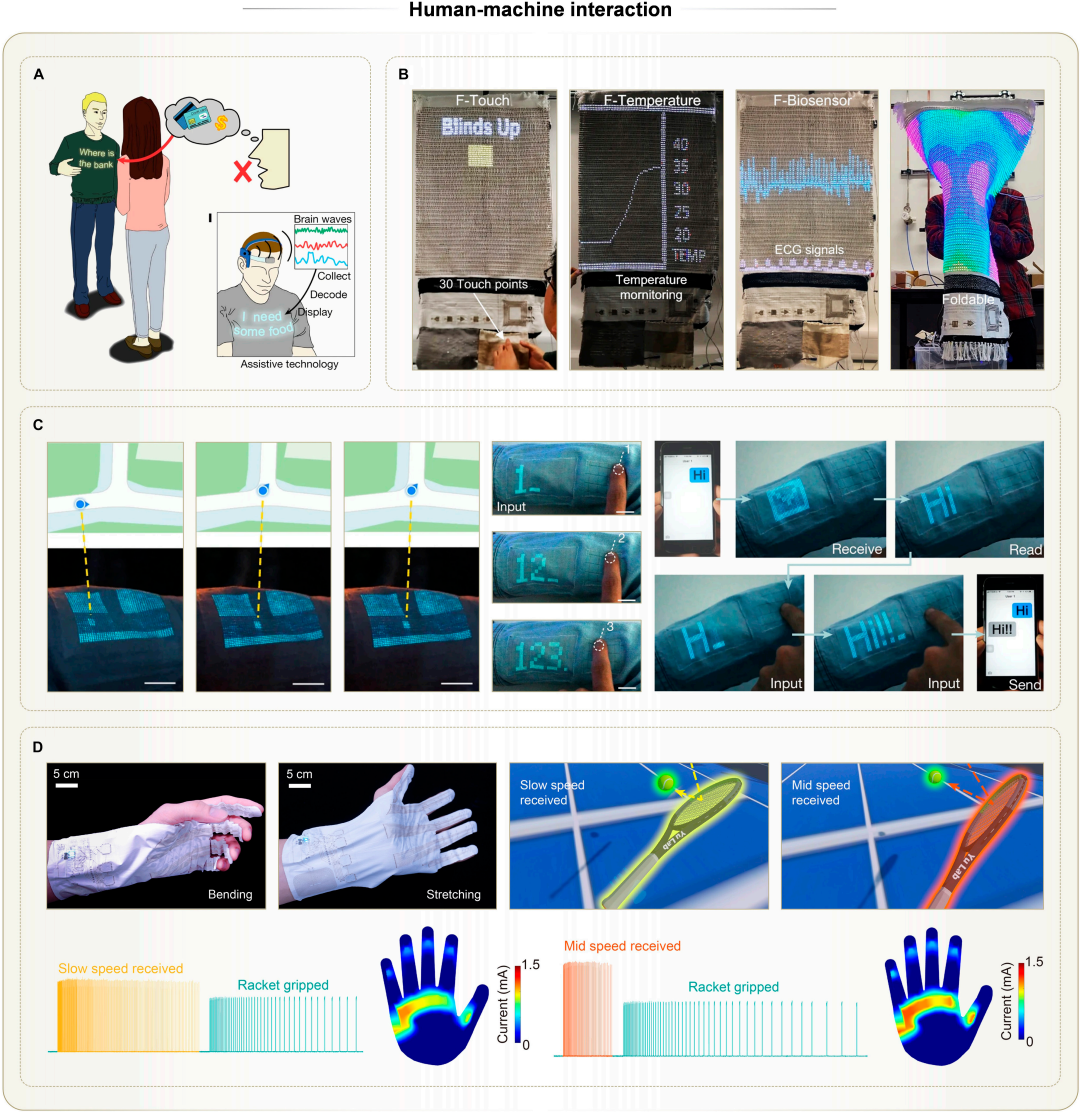
Applications of THE in human–machine interaction (part 1). (A) Schematic showing that the display textile enables communication via clothing. Reprinted with permission from [20]. Copyright 2021, Springer Nature. (B) Display textile constructed by embedding full-color LED chips. The textile is interfaced with various sensors, showing its visual responses to touch, temperature, ECG signals, as well as mechanical stability under folding. Reprinted with permission from [21]. Copyright 2022, Springer Nature. (C) Large-area display textile constructed by crossing electroluminescent fibers. Left: Real-time location displayed on a sleeve, which is synchronized with the location map on a smartphone. Scale bars, 1 cm. Middle: Text displayed on a sleeve, which is controlled by pressing the pressure sensors. Scale bars, 2 cm. Right: Receiving and sending messages between the integrated textile system and a smartphone. Reprinted with permission from [20]. Copyright 2021, Springer Nature. (D) Fully integrated haptic glove on nonwoven textiles with electrotactile feedback. Top left: Photos showing the ductility of the glove worn on a hand during bending and stretching. Top right: VR scenarios of a tennis game where dynamic feedback is delivered by the glove. Bottom left: Temporal dynamic feedback and the current distributions toward the hand during the game. Reprinted with permission from [74]. Copyright 2024, AAAS.

Vision accounts for over 80% of the information humans receive during interaction with external stimuli [134]. Integrating large-scale displays into textiles helps individuals with communication impairments to visually express information to their surroundings, thus promoting their participation in social collaboration (Fig. 10A) [20]. Researchers demonstrated a woven display textile by embedding small-sized and full-color LED chips into fibers [21]. By integrating additional functional fibers, this display was also equipped with wireless power transfer, touch sensing, and environmental and biological signal monitoring. Therefore, real-time sensory data and touch information were directly presented on the textile surface (Fig. 10B). However, the dense integration of rigid chips increased the system’s weight and stiffness. A more prospective solution involved constructing display pixels from cross-points of electroluminescent fibers, resulting in lighter and more flexible display textiles [20]. In such systems, the pixel spacing of ~800 μm enabled the display of fine text and punctuations (Fig. 10C). This ductile screen could visualize global positioning system (GPS) position and typed information. Future developments may focus on co-integrating driving circuits with the display textile itself, advancing toward real-world deployment.

#### Haptic interaction

Tactile sensing is another primary form of human interaction. Since hands are the most commonly used parts for tactile interaction, haptic gloves hold great potentials in this domain. Additionally, the high density of sweat glands on palms raises a demand for textile-based haptic gloves [135,136]. These gloves can offer superior air and moisture permeability, ensuring both comfort and reliable skin-machine interfaces. Using LM and LM hybrid solders on a stretchable nonwoven fabric, researchers developed a fully integrated haptic glove for electrotactile feedback in virtual environments [74]. The glove incorporated 128 independent electrodes and driving circuits, with each electrode connected to the skin via conductive hydrogels (Fig. 10D). When generating current pulses, the glove stimulated mechanoreceptors on hands, creating a highly realistic virtual touch sensation. In case studies, a volunteer held a virtual tennis racket and hit a tennis ball in a virtual reality (VR) environment. Meanwhile, the glove delivered real-time tactile feedback to the volunteer’s hands corresponding to virtual gripping and impacts. Due to the breathability of the glove, no sweat accumulation occurred at the skin–glove interface. This ensured the intimate bonding of hydrogels to the skin as well as crosstalk-free electrical stimulation.

**Fig. 11.**
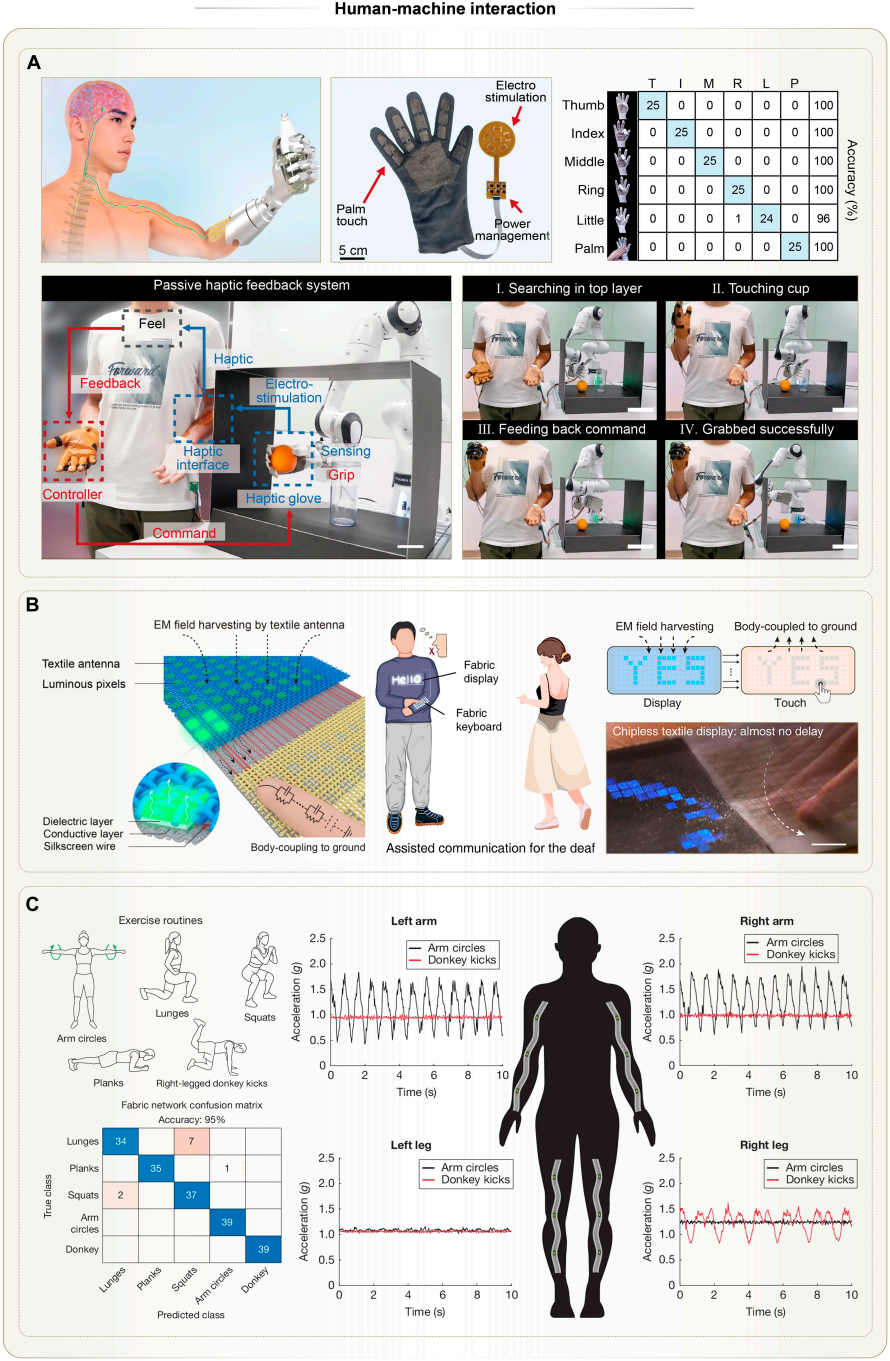
Applications of THE in human–machine interaction (part 2). (A) A self-powered textile haptic glove with tactile sensors and electrotactile feedback. Top left: Schematic showing the glove worn on a prosthetic hand, reconstructing the tactile sensation of an amputee by stimulating the mechanoreceptors on the forearm. Top middle: Photos showing embroidered tactile sensors on the gloves as well as the circuits for electrotactile feedback. Top right: Confusion matrix of recognizing different grip patterns using the glove. Bottom: Demonstration of the passive haptic feedback system, where the glove worn on the robotic hand can provide a sense of touch to the volunteer’s arm, allowing human control of the robotic hand through this sensory feedback. The cup in the black box is successfully grabbed. Reprinted with permission from [61]. Copyright 2025, AAAS. (B) Chipless fiber for both visual and tactile interactions. Left: Schematic showing the mechanism of a textile touch panel and a display textile made from this fiber. Middle: Schematic showing that the fiber provides assisted optical communication for the deaf. Right: Tactile patterned displays enabled by the textile touch panel and display textile, without batteries or chips in the system. Scale bar, 3 cm. Reprinted with permission from [71]. Copyright 2024, AAAS. (C) In-fiber computers distributed on limbs to infer motion patterns. Left top: Schematics of the 5 motion patterns during training. Left bottom: Confusion matrix showing the inferences made by 4 in-fiber computers on the wearer, exhibiting accuracy in inferring activities. Right: Representative plots of time-series accelerometer magnitude collected by each in-fiber computer when the training wearer performs arm circles and donkey kicks. Reprinted with permission from [35]. Copyright 2025, Springer Nature.

Beyond virtual tactile sensation, textile haptic gloves can enhance the perception of real-world objects. In one example, researchers embroidered Ag electrodes onto a woven glove and combined them with fluorinated ethylene propylene (FEP) films to form self-powered tactile sensors based on the TENG principle [61]. These sensors responded to physical contact and harvested energy for electrotactile stimulation (Fig. 11A). By distributing 6 TENG sensors across 5 fingers and the palm, the system not only discriminated grip patterns but also delivered corresponding tactile feedback to forearm mechanoreceptors through triboelectric discharge. When worn on a prosthetic hand, it transmitted tactile feedback from the prosthesis to the amputee’s residual limb, restoring complex tactile sensations. In demonstrations, the sensing part of the glove was mounted on a robotic hand, while the feedback module was connected to a human manipulator. This configuration allowed the user to perceive the relative position of the robotic hand and external objects, enabling precise grasping.

Furthermore, textile electronic systems can support multi-modal interaction involving both visual and tactile cues. One study developed a coaxial fiber consisting of an antenna core and a dielectric layer, which is capable of harvesting and storing electromagnetic energy from the contact with human body [71]. An outer optical layer responded to the stored electromagnetic energy and emitted light. Based on this principle, researchers constructed a 644-pixel textile touch panel paired with a matching display textile (Fig. 11B). When touched, the energy was stored in the corresponding touch panel pixel and transferred to the same location on the display via conductive wires. As a result, user-drawn gestures on the textile panel were instantly visualized on the display textile. Compared to other haptic and visual systems, this approach completely eliminated the local stiffness of embedded chips and the visual latency caused by computation.

#### Edge intelligence for distributed motion inference

In human–machine interaction scenarios, THE systems worn by users should not hinder natural movements. However, the local stiffness induced by concentrating numerous rigid electronic components in a single piece of textile tends to compromise user experience. Therefore, a distributed system layout is more conducive to comfortable and effective interactions. In such architectures, a promising opportunity lies in data fusion across distributed nodes. By leveraging information from different parts of the body, the system can infer complex behaviors. To enable this data fusion, both intra-node data preprocessing and inter-node data transmission should be addressed. Using thermal drawing and 3D interposer technologies, researchers integrated a 3-axis accelerometer, a microcontroller, and a BLE module into a single drawn fiber computer [35]. The accelerometer collected motion data, the microcontroller executed a locally deployed neural network for data preprocessing, and the BLE was used to transmit data from all nodes to a central node for fusion. Four such fibers were distributed on each limb of a user to monitor motion patterns (Fig. 11C). Owing to the in-fiber integration architecture and the distributed system layout, the user maintained full mobility during movements. By fusing data from the 4 fibers, the system achieved a 95% classification accuracy across 5 body postures, demonstrating its strong potential for motion-driven human–machine interaction.

At the current stage, examples of edge intelligence in textiles are rare, as they require the seamless integration of both sensors and chips without hindering natural human movements. More examples are expected to emerge in the near future.

## Conclusions and Outlooks

### Future architectures of THE

The permeability of textile electronic systems provides pronounced advantages for continuous use in sweating body areas. Beyond comfort, system-level functionality becomes a key differentiator in next-generation textile electronics. For this purpose, versatile electronic components are integrated in one system, including flexible components with specific functions as well as rigid components for general-purpose processing.

Compared with conventional textile electronic systems, the critical superiority of THE lies in the seamless integration of signal conditioning and computation. This offers tremendous potentials for the combination of AI, Internet of Things (IoT) technologies, and textile electronics [137]. On the one hand, THE with built-in signal processing eliminates the need for external circuit boards, contributing to enhanced wearing comfort. This comfort facilitates continuous data collection throughout long-term human activities, thus providing large-scale data support for the training of AI algorithms [138]. On the other hand, such sensing systems can function as distributed nodes across the body, forming a sensor network. Each subnode is capable of performing algorithms, while a central node aggregates the results and carries out higher-level inferences [139]. Therefore, distributed architectures with edge intelligence represent a key development trend for future THE.

To achieve distributed and intelligent textile electronic systems, THE systems with different form factors should be utilized in conjunction. For in-fiber THE, it offers the highest degree of wearing comfort and substrate compatibility [40]. Although it has been extended to a growing variety of chips, its circuit architecture remains limited because the chips within a single fiber are inherently connected in parallel. Additionally, in-fiber THE only supports chips of limited sizes. Therefore, this method is better suited for highly customized and simpler tasks as distributed nodes. For complex and general-purpose circuitry in a central node, LBL integration is the preferred approach. LBL THE allows freeform circuit layouts and the integration of larger chips [72]. It is evolving toward high chip density and overall stretchability, gradually approaching the performance of thin-film hybrid electronics [28].

**Fig. 12.**
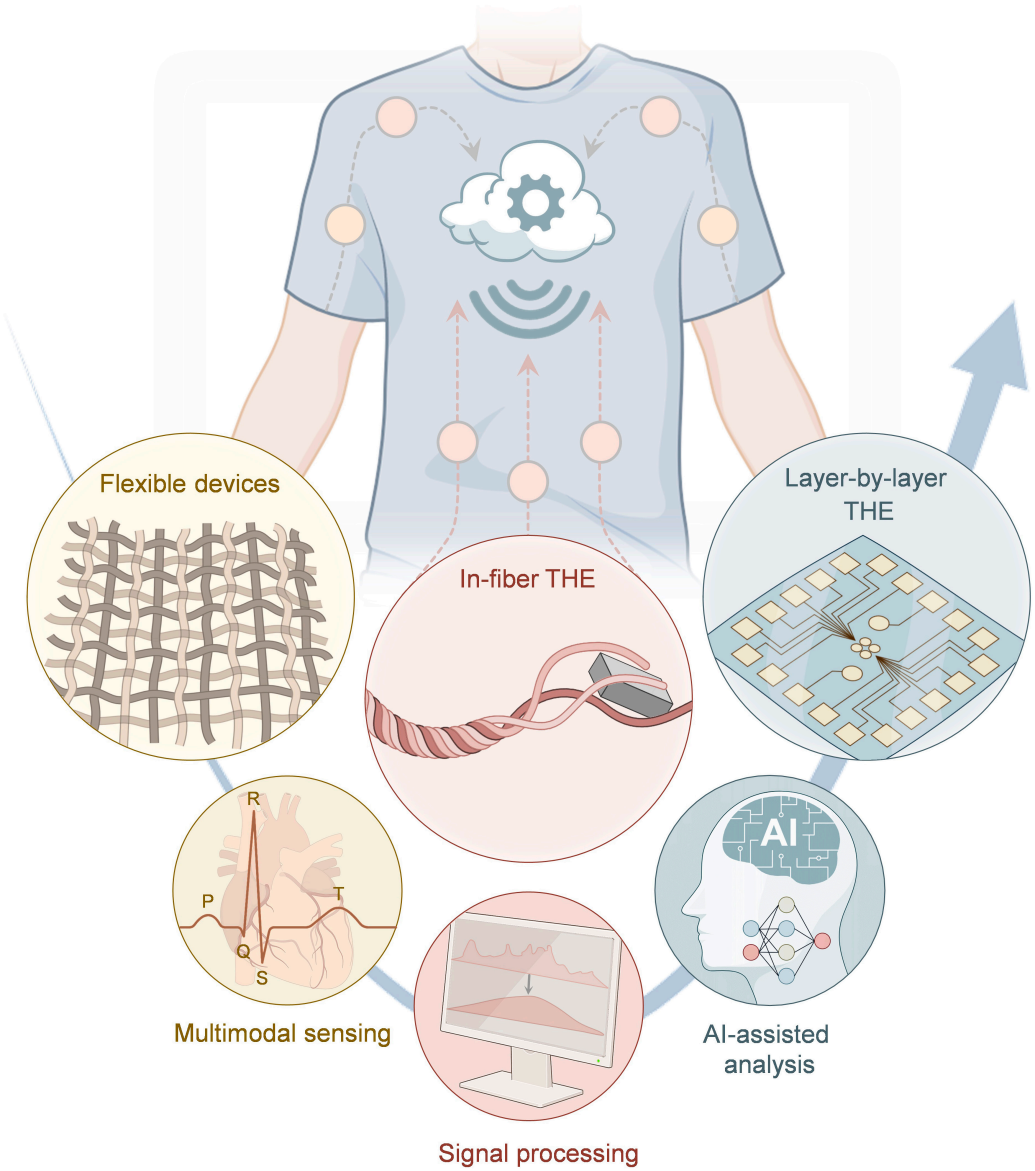
Future architectures of THE. The combination of LBL THE, in-fiber THE, and diverse flexible electronic components forms a distributed architecture with edge intelligence. Some figure elements were created using BioRender.com and Figdraw.com.

We envision that the ideal THE architecture should involve a ternary combination of LBL THE, in-fiber THE, and various flexible electronic components (Fig. 12): (a) Flexible components directly interact with human body and environments, performing sensing, actuation, luminescence, energy storage, and capturing. (b) In-fiber THE conducts edge computing via embedded chips. It can filter and digitize sensory signals, generate control signals, store and infer data, and finally aggregate results to the central hub. In addition, in-fiber THE can be functionalized with various micro-electro-mechanical systems (MEMS) chips, such as MEMS sensors and actuators. (c) LBL THE serves as the central hub, coordinating tasks and interacting with cell phones or laptops.

### Challenges and potential solutions

**Fig. 13.**
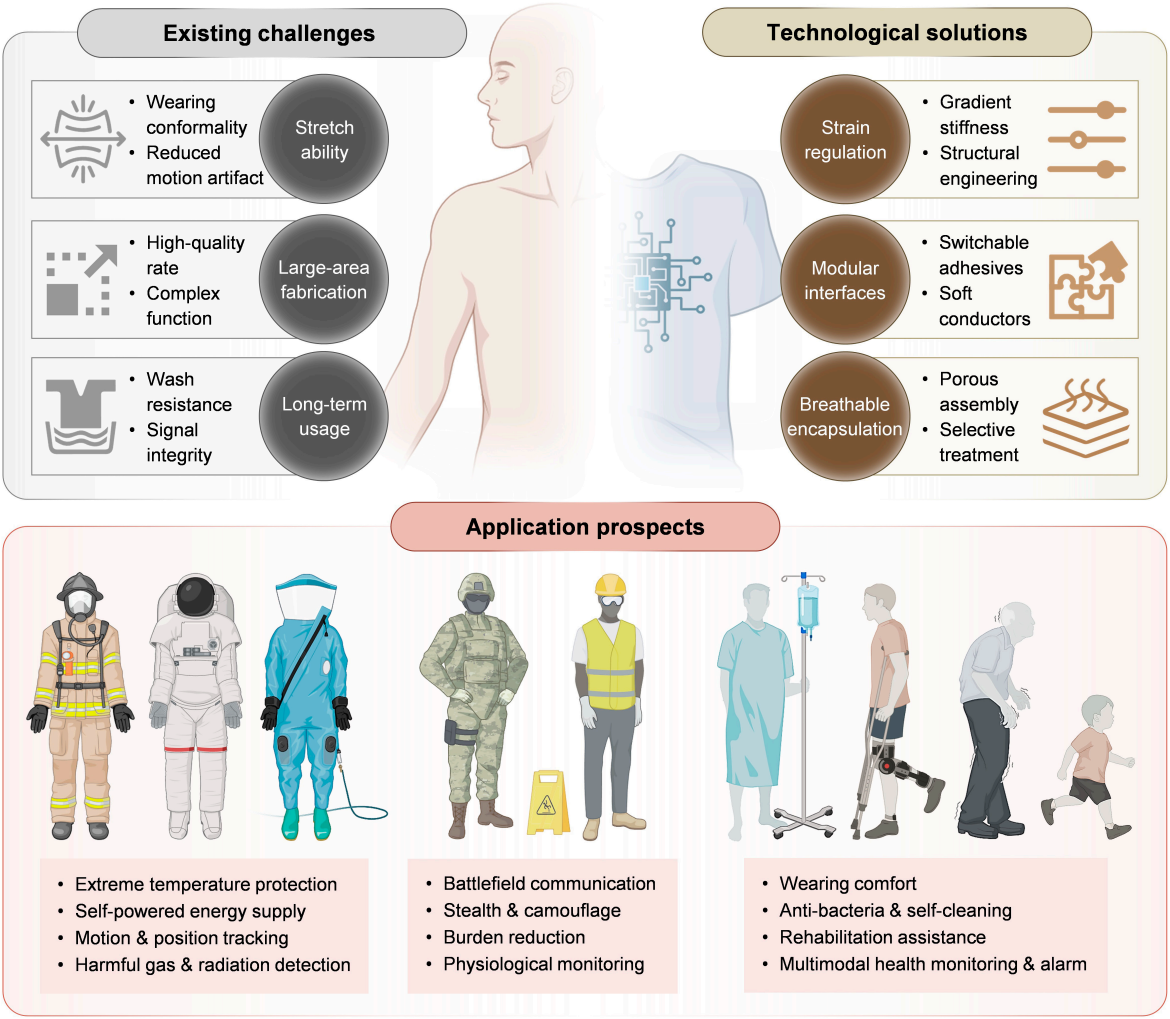
Existing challenges, technological solutions, and application prospects of THE. Some figure elements were created using BioRender.com.

To date, seamless integration between THE and diverse flexible textile components remains unexplored (Fig. 13). Existing challenges include the following: (a) Relatively low stretch stability: Stretch stability at the heterogeneous interfaces of wearable electronics is crucial for wearing conformality and low motion artifacts [140,141]. In THE, modulus mismatches between rigid electronics and soft textiles, or between different textile substrates, impair electrical stability under stretch. (b) Large-area fabrication: Manual operations during the fabrication of THE introduce inconsistencies, leading to lower quality rates, particularly when fabricating large-scale systems [23]. Furthermore, the current size of LBL THE systems cannot accommodate the complex architecture required to drive high-density textile electronics, such as display textiles [20]. (c) Long-term usage: Textile electronic components in direct contact with skin are subject to staining and corrosion from sweat, which is bound to degrade signal integrity over time. While washing is essential to remove these adverse effects, most LBL THE systems are not competent enough to withstand machine washing [142]. For the long-term usage of textile electronic systems, their washability should be enhanced. However, the reported methods for washable textile electronic systems often compromise permeability or circuit density, with inevitable mechanical failures after tens of wash cycles [127].

To address these limitations, potential solutions are as follows (Fig. 13): (a) Strain regulation: By designing textile substrates with gradient stiffness or island-bridge architectures, strain can be confined to noncritical regions, preserving the integrity of heterogeneous interfaces during stretching [143]. This design imparts global stretch stability to THE systems. Moreover, effective strain regulation is expected to enhance their washability. This is because the connections between rigid electronic modules and stretchable interconnects are particularly vulnerable to mechanical stresses during washing. The concept of strain regulation has been extensively applied in thin-film hybrid electronics and now requires applicable manufacturing methods for textile electronics. (2) Modular interfaces: Decomposing a heterogeneous textile electronic system into separate modules benefits both large-scale production and long-term maintainability [144,145]. From the manufacturing perspective, electronic modules with simpler constructions are easier to fabricate than a monolithic system. By separately fabricating and subsequently assembling heterogeneous modules, such as fully flexible textile electronic components, in fiber THE and LBL THE, it becomes feasible to realize large-scale systems that would be impractical to produce as a whole. Considering the maintainability, modular THE systems allow instant replacement of defective or degraded modules, without compromising system functionality. Furthermore, modules with different levels of washability can be disassembled and washed separately before being reassembled. Therefore, LBL THE modules in a garment can be washed less frequently than other flexible or in-fiber THE modules. The overall wash lifespan of modular THE systems is expected to exceed that of monolithic counterparts. The modular architecture necessitates detachable interfaces that can maintain robust conductivity across multiple cycles. Switchable adhesives show promise as backbone materials for such interfaces due to their mechanical robustness and easy detachability [146]. Additionally, soft conductive materials are essential for establishing reliable conductive paths between modules [147]. (3) Breathable encapsulation: Encapsulation provides protection against contaminants and mechanical damage, thereby supporting long-term use. For breathable textiles, the encapsulation itself must also be permeable. This can be achieved via electrospun porous fiber mats [73] or selective coating methods targeting only the electronics. Ideally, such encapsulation should be replaceable to prevent pore clogging by dust accumulation.

Looking ahead, THE holds great promise for integrating advanced electronic functionalities into everyday textile platforms, bridging the gap between wearable electronics and daily life. The future of THE will feature diverse sensing modalities, seamless human–machine interactions, and other innovative technologies. These advances will unlock impactful applications in daily scenarios and high-risk occupations, as well as assistance for vulnerable populations (Fig. 13). For instance, protective garments integrated with THE can provide real-time physiological and environmental monitoring for firefighters, astronauts, and disinfection personnel. Similarly, soldiers and construction workers may benefit from improved situational awareness and safety through sensing systems embedded in their garments. In healthcare, patient gowns and daily wear for elderly individuals or children can be equipped with imperceptible sensors to support continuous health monitoring, fall detection, and emergency alerts. These examples highlight the transformative potentials of THE across domains requiring intelligence and wearing comfort.
